# Associations Between Gut Microbiota and Immune Profiles in Community‐Dwelling Older Adults and the Regulatory Role of *Polygonatum odoratum*

**DOI:** 10.1111/acel.70724

**Published:** 2026-09-23

**Authors:** Tingting Xia, Hongmei Wu, Tianyi Cui, Wenzhi Yang, Min Zhang, Yuhong Huang, Shengyuan Zhou, Yongsheng Wu, Donglan Yan, Fan Jia, Lin Zhang, Zhi Cui, Jiayao An, Xinqin Zhong, Bin Lv, Xin Zhao, Kaijun Niu, Xiumei Gao

**Affiliations:** ^1^ Ministry of Education Key Laboratory of Pharmacology of Traditional Chinese Medical Formulae Tianjin University of Traditional Chinese Medicine Tianjin China; ^2^ State Key Laboratory of Chinese Medicine Modernization Tianjin University of Traditional Chinese Medicine Tianjin China; ^3^ School of Public Health Tianjin University of Traditional Chinese Medicine Tianjin China; ^4^ Preventive Treatment Department Second Affiliated Hospital of Tianjin University of Traditional Chinese Medicine Tianjin China; ^5^ Jiuzhitang Co. Ltd Changsha Hunan China; ^6^ Division of Biostatistics, School of Public Health UT Health Science Center Houston Texas USA; ^7^ First Teaching Hospital of Tianjin University of Traditional Chinese Medicine, National Clinical Research Center for Chinese Medicine Acupuncture and Moxibustion Tianjin China

**Keywords:** aging, gut microbiota, immunosenescence, *Polygonatum odoratum*

## Abstract

Aging is often associated with intestinal microbiota imbalance and immune system degeneration. The dysregulation and crosstalk of these two factors markedly elevate the elderly's susceptibility to infections and age‐related diseases. *Polygonatum odoratum*, a traditional Chinese medicinal herb, is widely used in geriatric care for its lung‐moistening and nourishing effects. However, its potential regulatory roles on the microbiota and immunity in the aging context remain unreported. A total of 140 independently living elderly individuals aged 60 and above were recruited for this study. The randomized, double‐blinded study including YZG (*P. odoratum*) and placebo groups was performed for 90 days' intervention. Peripheral blood immune cell subsets were analyzed by flow cytometry, and microbial diversity in fecal samples was assessed by 16S rRNA sequencing. Baseline correlation analysis revealed that alterations in gut microbiota were predominantly associated with T cell subsets. Specifically, *Akkermansia* showed a significant negative correlation with age‐related memory T cells, including CD4^+^ and CD8^+^ TEMRA. Additionally, *Klebsiella* exhibited a significant positive correlation with partial subsets of CD4^+^ TEMRA. After 90‐day YZG intervention, the percentage of TEMRA and CD28^−^ CD57^+^ TEMRA cells were reduced in elderly subjects. The relative abundance of *Prevotella 9* and *Akkermansia* were increased, and the abundance of *Klebsiella* in the gut microbiota were significantly decreased. Correlation analysis linked YZG‐induced gut microbiota changes to improved immunity and fewer respiratory infections. This study demonstrates significant correlations that exist between immunosenescence in T‐cell compartments and gut microbial composition, suggesting a potential interplay between microbial health and immune aging.

**Trial Registration:** UMIN Clinical Trials Registry identifier: UMIN000052572

## Graphical abstract

Gut Microbiota‐Immune Associations in Community‐Dwelling Older Adults: Role of *Polygonatum odoratum*.

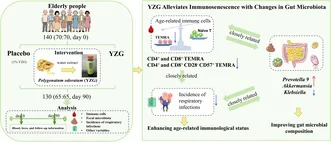

**Abbreviations**
CD4^+^ TCM: CD4^+^ central memory T cell
CD4^+^ TEM: CD4^+^ effector memory T cell
CD4^+^ TEMRA: CD4^+^ terminally differentiated effector memory T cells re‐expressing CD45RA
CD8^+^ TCM: CD8^+^ central memory T cell
CD8^+^ TEM: CD8^+^ effector memory T cell
CD8^+^ TEMRA: CD8^+^ terminally differentiated effector memory T cells re‐expressing CD45RA
HDL‐CH: high‐density lipoprotein cholesterol
IBD: inflammatory bowel disease
UA: blood uric acid
YZG: Yuzhu Gao

## 1 Introduction

The aging process is accompanied by an age‐dependent decline in immune system function, known as immunosenescence, the most prominent feature of which is the aging of T cells (Wang et al. 2022). With aging, the number of naïve T cells decreases, whereas memory T cells increase. Notably, the accumulation of terminally differentiated memory T cells re‐expressing CD45RA (TEMRA) is particularly prominent and exhibits typical features of cellular senescence, such as telomere shortening, an enhanced senescence‐associated secretory phenotype, and cell cycle arrest (Lance et al. 2026). They not only occupy the immunological niche but also exacerbate the chronic inflammatory environment and impair the clearance of pathogens. Particularly, promoting the clearance and accumulation of terminally differentiated memory T cells CD57^+^ CD28^−^ subset represents an important strategy for delaying the onset and progression of age‐related diseases (Salumets et al. 2022).

The aging process is often accompanied by an imbalance in intestinal microecological homeostasis, primarily characterized by reduced microbial diversity, excessive colonization by harmful bacteria, and decreased abundance of beneficial bacteria. These alterations can compromise intestinal barrier integrity, impair the body's immune function, and subsequently drive the onset and progression of chronic inflammation, neuroinflammation, metabolic syndrome, gut‐brain axis dysregulation, and neurodegenerative diseases (Gu et al. 2025; Liang et al. 2026). Comparative studies indicate that, relative to elderly high‐level orienteering athletes who experience mild gastrointestinal symptoms and report higher life satisfaction, the general elderly population exhibits a significantly lower abundance of beneficial bacteria such as *Akkermansia muciniphila* and *Faecalibacterium prausnitzii* in their intestines (Schoultz et al. 2025). Reconstructing and maintaining intestinal microbiota homeostasis is a necessary strategy for promoting healthy aging in the elderly population.

Exploring the link between the gut microbiota and the immune system has emerged as a critical therapeutic target in aging and related disease management (Ding et al. 2025). Several clinical studies demonstrated that probiotic intervention modulated systemic immune markers in patients with syndrome, while another cohort study revealed that specific gut microbial signatures were closely correlated with immune profiles in elderly populations (Zhu et al. 2024). The community‐dwelling older adults—a population in which such attention is often lacking before the onset of clinical disease (Colica and Vecchio 2026), often indulge in immune‐boosting herbal remedies, hailed as panaceas, yet blindly consume them without grasping their true effects—a testament to the gap between tradition and scientific understanding. Polygonati Odorati Rhizoma (*Polygonatum odoratum*, Yuzhu in Chinese) is a representative of the bodybuilding and *yin*‐nourishing herbs. *P. odoratum* is clinically used for elderly people in the prevention and treatment of respiratory tract infection and the related symptoms (Nan et al. 2023). It shows good pharmacological activities including anti‐inflammatory, antioxidant, hypoglycemic and lipid‐lowering, immune regulation, anti‐aging, regulating gut microbiota, and reducing obesity (Zhao et al. 2018). Its active components mainly include polysaccharides, saponins, flavonoids, anthraquinones, lectins, and other compounds (Zhao et al. 2019). However, the underlying boosting mechanism of *P. odoratum* on immunosenescence and gut microbiot in the elderly population is still unclear.

In this study, we first investigated the associations between the gut microbiota and immune cell subsets in a primary elderly population composed of community‐dwelling older adults. Subsequently, through an intervention trial, the effects of *P. odoratum* on reshaping host immune homeostasis as indicated by peripheral blood immune cell subsets and modulating the fecal microbial composition were systematically evaluated. We aim to provide a scientific foundation for elucidating the association between gut microbiota and immunity in community‐dwelling older adults, and for evaluating the interventional effects of commonly used supplements, such as *P. odoratum*.

## 2 Methods

### 2.1 Study Design

We recruit the elderly people in community of Tianjin and designed a double‐blind randomized controlled trial, which has been registered with the UMIN Clinical Trials Registry (Identifier: UMIN000052572). Ethical approval was given by the Research Ethics Committee of the Second Affiliated Hospital of Tianjin University of Chinese Medicine (Project No. EC.AT/03.19‐01/10.0) and was conducted in accordance with the guidelines set out in the Declaration of Helsinki. All the participants gave written informed consent.

### 2.2 Study Recruitment and Participants

**FIGURE 1:**
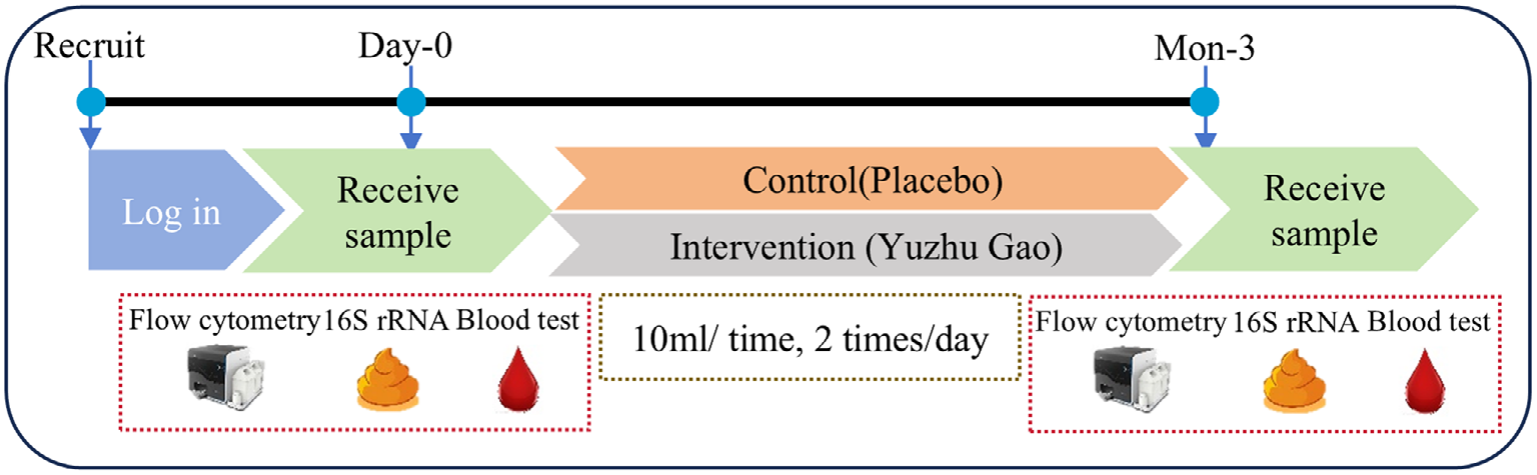
Intervention protocol.

A total of 186 participants were recruited by the physician from three communities between November 2023 and March 2024 in Tianjin, China, with the selection process described in Figure 1. Inclusion criteria were as follows: (1) age ≥ 60 years; (2) no communication and intellectual disabilities, able to live independently; (3) long term residence in Tianjin; (4) agreement to study follow‐up. Exclusion criteria include: (1) age < 60 years; (2) patients with severe depression, severe dementia, and visual or hearing impairment; (3) severe cardiovascular and cerebrovascular diseases (coronary heart disease, stroke, and myocardial infarction, etc.), tumors, liver and kidney diseases, etc.; (4) have a history of asthma, allergies, or allergies; (5) cannot complete the follow‐up.

### 2.3 Randomization and Masking

**FIGURE 2:**
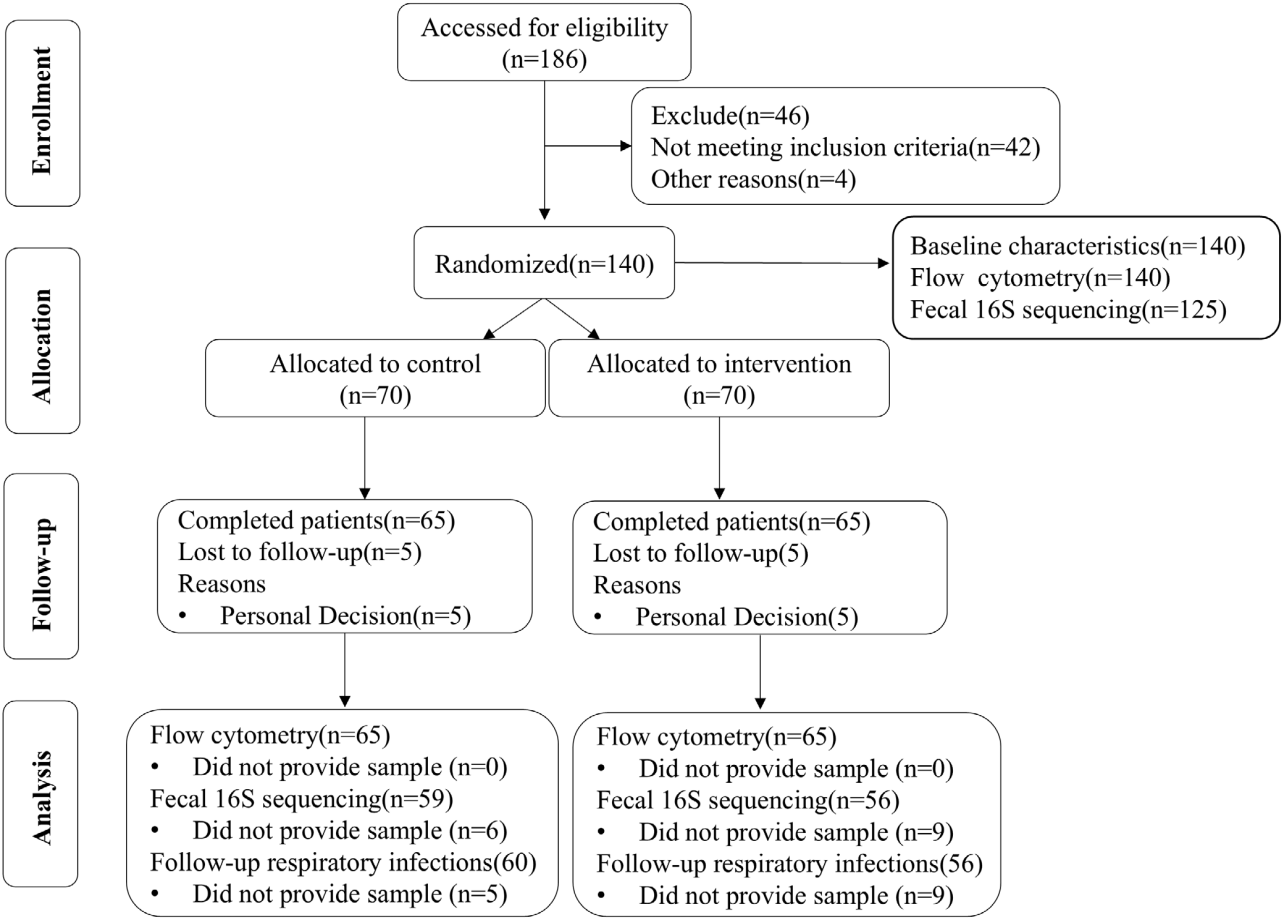
Flow diagram of enrollment, randomization, and follow‐up.

The elderly people in the study population were randomized into intervention group and control group in a 1:1 ratio. The double‐blind experiment was used, and neither the elderly participants nor the researchers could know the specifics of the group until the end of the clinical trial. The follow‐up process is depicted in Figures 1 and 2.

### 2.4 Intervention

Both Yuzhu Gao (YZG) for YZG group and placebo (Con) for control group used in the study were provided by Jiuzhitang Co. Ltd. (Lot No. 041A‐2203001‐11, China). YZG is a 100% aqueous extract of *Polygonatum odoratum* (Mill.) Druce, which is prepared by extracting the raw material with water three times, followed by concentration, standing, filtration, and final packaging. YZG and placebo are the same texture and adopt the same packaging and specifications.

The polysaccharide content as quality control of YZG was extracted and detected with multi‐level fingerprinting (Liu et al. 2024). An ultra‐high‐performance liquid chromatography‐Q exactive hybrid quadrupole orbitrap high‐resolution accurate mass spectrometer (Vion IMS‐QTOF) analysis was performed for the non‐polysaccharide components dection (Figure S1 and Table S1).

### 2.5 Assessments of Clinical Outcomes

Peripheral blood samples and fecal samples were collected at day 0 and the end of the intervention (day 90). Immune cell parameters in peripheral blood were examined by flow cytometry (BD FACSAria III flow cytometry, BD Biosciences) and antibodies were purchased from Biolegend (antibody labeling: Table S2 and circle gate strategy: Figure S2). Fecal gut microbiome composition was measured by V3‐V4 regions of 16S rRNA sequencing (LC‐Bio Technology Co. Ltd., Hangzhou, China). Blood immune markers and gut microbiome were the primary outcomes of this study, while respiratory infection incidence and other clinical symptom ratings were secondary outcomes. There was no adverse event throughout the intervention period. Detailed methodological information and other relevant variables are described in the Materials and Methods section in the Supporting Information.

### 2.6 Statistical Analysis

Statistical analysis was performed using GraphPad Prism 8.0.1, with qualitative data expressed as percentages (%) and analyzed by Chi‐square test, while quantitative data were presented as Mean ± SD (analyzed by ANOVA/*t*‐test for normally distributed data) or median and IQR (analyzed by Kruskal–Wallis/Mann–Whitney test for non‐normally distributed data), using two‐sided tests with *p* < 0.05 considered significant. 16S rRNA data were analyzed using R‐4.4.2: alpha diversity (violin plots, Wilcoxon test), beta diversity (PCoA, PERMANOVA), species abundance (Kruskal–Wallis/ANCOM‐BC), and microbiota‐immunity correlations (Spearman). The detailed information can be seen in Materials and Methods (Supporting Information).

## 3 Results

### 3.1 General Information of Participants

The baseline characteristics of the two groups (*n* = 70) were roughly the same between our YZG and placebo groups (Table 1). Individuals who completed the trial (*n* = 65) were included in the endpoint analysis. In addition, ITT analysis was performed for all 140 individuals by replacing the missing data with the last observed value.

**TABLE 1:** Baseline characteristics.

	Con group	YZG group	*p*
Age, years	68.0 (66.00, 73.00)	71.0 (63.75, 75.00)	0.966
Gender			0.858
Male, *n* (%)	24 (34.3)	23 (32.9)	
Female, *n* (%)	46 (65.7)	47 (67.1)	
BMI, kg/m^2^	25.20 (±3.518)	25.20 (±3.438)	0.996
Education level ≥ high school, *n* (%)	45 (72.6)	40 (63.5)	0.276
Non‐solitary, *n* (%)	54 (87.1)	49 (77.8)	0.171
Household income ≥ 5000 (RMB/Mon), *n* (%)	47 (75.8)	40 (66.7)	0.271
Drinking alcohol, *n* (%)			0.638
Drink every day	2 (2.9)	4 (5.7)	
Drink occasionally	15 (21.4)	21 (30.0)	
Used to drink	5 (7.1)	5 (7.1)	
Never drink	40 (57.1)	33 (47.1)	
Drinking no answer	8 (11.4)	7 (10.0)	
Smoking, *n* (%)			0.061
Smoking	1 (1.4)	8 (11.4)	
Used to smoke	10 (14.3)	5 (7.1)	
Never smoked	50 (71.4)	50 (71.4)	
Smoking no answer	9 (12.9)	7 (10.0)	

### 3.2 Blood Routine, Liver and Kidney Function and Lipid Profile

The results of blood tests are shown in Table S3. After intervention in the YZG group, there was a significant increase in glycosylated hemoglobin, serum albumin, high‐density lipoprotein cholesterol, and serum total protein, while the mean corpuscular hemoglobin concentration, mean corpuscular hemoglobin, and uric acid levels decreased significantly. No significant differences were observed in the remaining blood test parameters.

### 3.3 Relationships Between Blood Immune Cell Subsets and Gut Microbiota Profiles and in the Elderly

**FIGURE 3:**
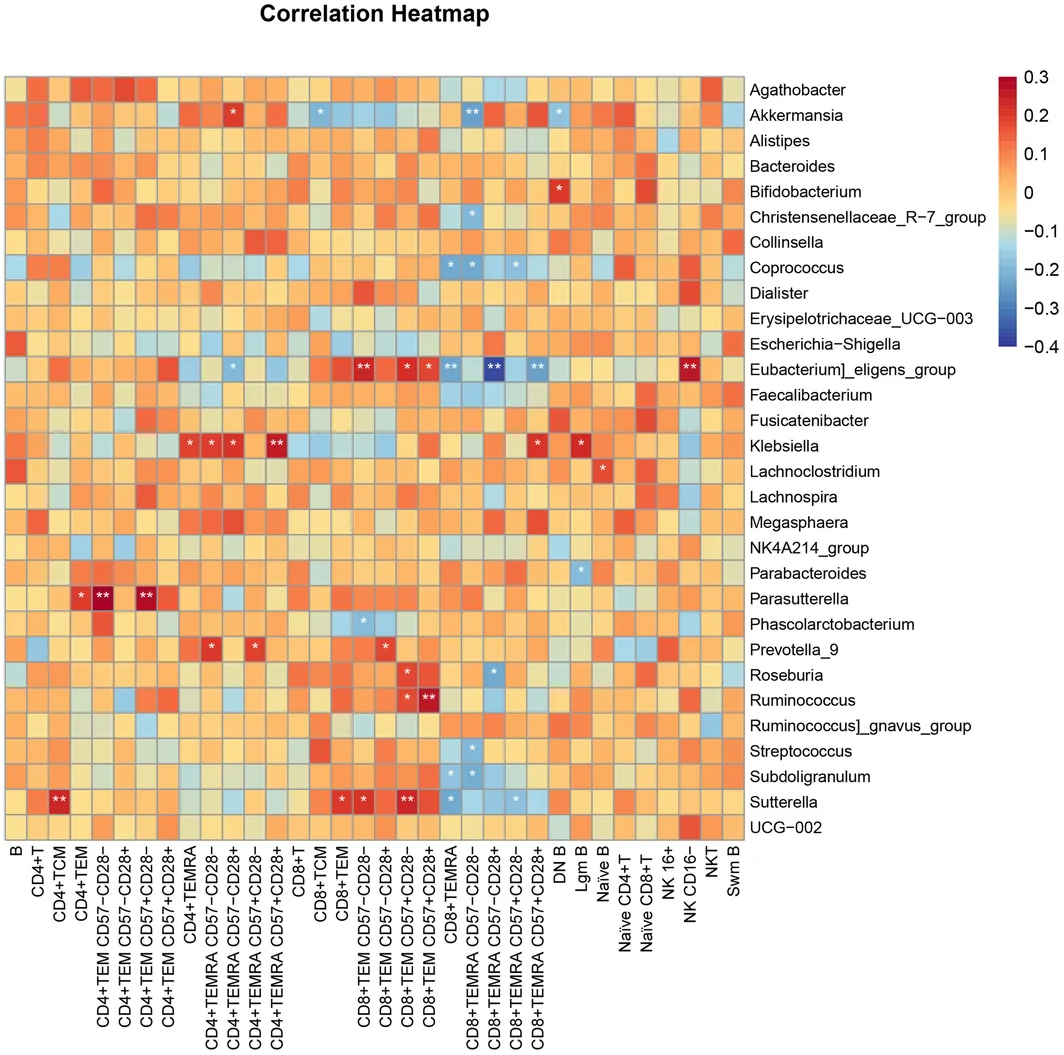
Heatmap of correlation analysis between gut microbiota and immune cells in the baseline population. *p < 0.05, **p < 0.01.

At baseline, correlation analysis was performed between gut microbiota and immune cell subsets in a cohort of 125 elderly individuals. The results revealed that alterations in gut microbiota were predominantly associated with T cell subsets. *Akkermansia* was significantly negatively correlated with the CD8^+^ terminally differentiated effector memory T cell (TEMRA) CD57^−^ CD28^−^ (*r* = −0.244, *p* = 0.006), CD8^+^ central memory T cells (TCM) (*r* = −0.205, *p* = 0.022), and double‐negative B cells (DN B) (*r* = −0.244, *p* = 0.044), while it was significantly positively correlated with the CD4^+^ TEMRA CD57^−^ CD28^+^ (*r* = 0.197, *p* = 0.027) and naïve CD4^+^ T cells. *Bifidobacterium* showed a significant positive correlation with DN B cells (*r* = 0.205, *p* = 0.022). *Christensenellaceae_R‐7_group* was significantly negatively correlated with the CD8^+^ TEMRA CD57^−^ CD28^−^ (*r* = −0.197, *p* = 0.028). *Coprococcus* was significantly negatively correlated with CD8^+^ TEMRA (*r* = −0.227, *p* = 0.011), the CD8^+^ TEMRA CD57^−^ CD28^−^ (*r* = −0.223, *p* = 0.012), and the CD8^+^ TEMRA CD57^+^ CD28^−^ subset (*r* = −0.187, *p* = 0.036). *The Eubacterium eligens group* demonstrated significant negative correlations with CD8^+^ TEMRA CD57^−^ CD28^+^ (*r* = −0.370, *p* = 2.142), CD8^+^ TEMRA CD57^+^ CD28^+^ (*r* = −0.238, *p* = 0.008), CD8^+^ TEMRA (*r* = −0.233, *p* = 0.009), and CD4^+^ TEMRA CD57^−^ CD28^+^ (*r* = −0.196, *p* = 0.028). Conversely, it showed significant positive correlations with NK CD16^−^ (*r* = 0.263, *p* = 0.003), CD8^+^ TEM CD57^−^ CD28^−^ (*r* = 0.234, *p* = 0.009), CD8^+^ TEM CD57^+^ CD28^−^ (*r* = 0.218, *p* = 0.015), and CD8^+^ TEM CD57^+^ CD28^+^ (*r* = 0.186, *p* = 0.038). *Parabacteroides* was significantly negatively correlated with long‐lived memory B cells (Lgm B) (*r* = −0.189, *p* = 0.035). *Phascolarctobacterium* was significantly negatively correlated with the CD8^+^ effector memory T cell (TEM) CD57^−^ CD28^−^ (*r* = −0.193, *p* = 0.030). *Streptococcus* was significantly negatively correlated with the CD8^+^ TEMRA CD57^−^ CD28^−^ (*r* = −0.198, *p* = 0.027). *Subdoligranulum* was significantly negatively correlated with the CD8^+^ TEMRA CD57^−^ CD28^−^ (*r* = −0.219, *p* = 0.014) and CD8^+^ TEMRA (*r* = −0.181, *p* = 0.044). *Sutterella* was significantly negatively correlated with CD8^+^ TEMRA (*r* = −0.224, *p* = 0.012) and the CD8^+^ TEMRA CD57^+^ CD28^−^ (*r* = −0.184, *p* = 0.040). A significant negative correlation was observed between *Roseburia* and CD8^+^ TEMRA CD57^−^CD28^+^ cells (*r* = −0.227, *p* = 0.011), while a significant positive correlation was identified with CD8^+^ TEM CD57^+^ CD28^−^ (*r* = 0.183, *p* = 0.042). *Sutterella* exhibited significant negative correlations with CD8^+^ TEMRA (*r* = −0.224, *p* = 0.012) and CD8^+^ TEMRA CD57^+^ CD28^−^ (*r* = −0.184, *p* = 0.040). Positive correlations were found between *Sutterella* and CD8^+^ TEM CD57^+^ CD28^+^ (*r* = 0.234, *p* = 0.009), CD4^+^ TCM (*r* = 0.231, *p* = 0.009), CD8^+^ TEM CD57^−^ CD28^−^ (*r* = −0.209, *p* = 0.019), and CD8^+^ TEM cells (*r* = 0.204, *p* = 0.023). *Klebsiella* was positively correlated with CD4^+^ TEMRA CD57^+^ CD28^+^ (*r* = 0.257, *p* = 0.004), IgM^+^ B cells (*r* = 0.224, *p* = 0.012), CD8^+^ TEMRA CD57^+^ CD28^+^ (*r* = 0.209, *p* = 0.019), CD4^+^ TEMRA CD57^−^ CD28^+^ (*r* = 0.209, *p* = 0.020), CD4^+^ TEMRA CD57^−^CD28^−^ (*r* = 0.120, *p* = 0.025), and CD4^+^ TEMRA cells (*r* = 0.188, *p* = 0.036). *Lachnoclostridium* showed a positive correlation with naïve B cells (*r* = 0.177, *p* = 0.048). *Parasutterella* was positively correlated with CD4^+^ TEM CD57^−^ CD28^−^ (*r* = 0.304, *p* = 0.001), CD4^+^ TEM CD57^+^ CD28^−^ (*r* = 0.283, *p* = 0.001), and CD4^+^ TEM cells (*r* = 0.201, *p* = 0.025). *Prevotella 9* demonstrated positive correlations with CD4^+^ TEMRA CD57^−^ CD28^−^ (*r* = 0.201, *p* = 0.024), CD4^+^ TEMRA CD57^+^ CD28^−^ (*r* = 0.188, *p* = 0.034), and CD8^+^ TEM CD57^−^ CD28^+^ cells (*r* = 0.182, *p* = 0.042). *Ruminococcus* was positively correlated with CD8^+^ TEM CD57^+^ CD28^+^ (*r* = 0.264, *p* = 0.003) and CD8^+^ TEM CD57^+^ CD28^−^ (*r* = 0.177, *p* = 0.048). Specifically, *Akkermansia* demonstrated a significant negative correlation with partial subsets of CD8^+^ TEMRA, and positive correlation with naïve CD4^+^ T. Additionally, *Klebsiella* exhibited a significant positive correlation with partial subsets of CD4^+^ TEMRA and CD8^+^ TEMRA (Figure 3).

### 3.4 Yuzhu Gao Alters Blood Immune Cell Subsets in the Elderly

**FIGURE 4:**
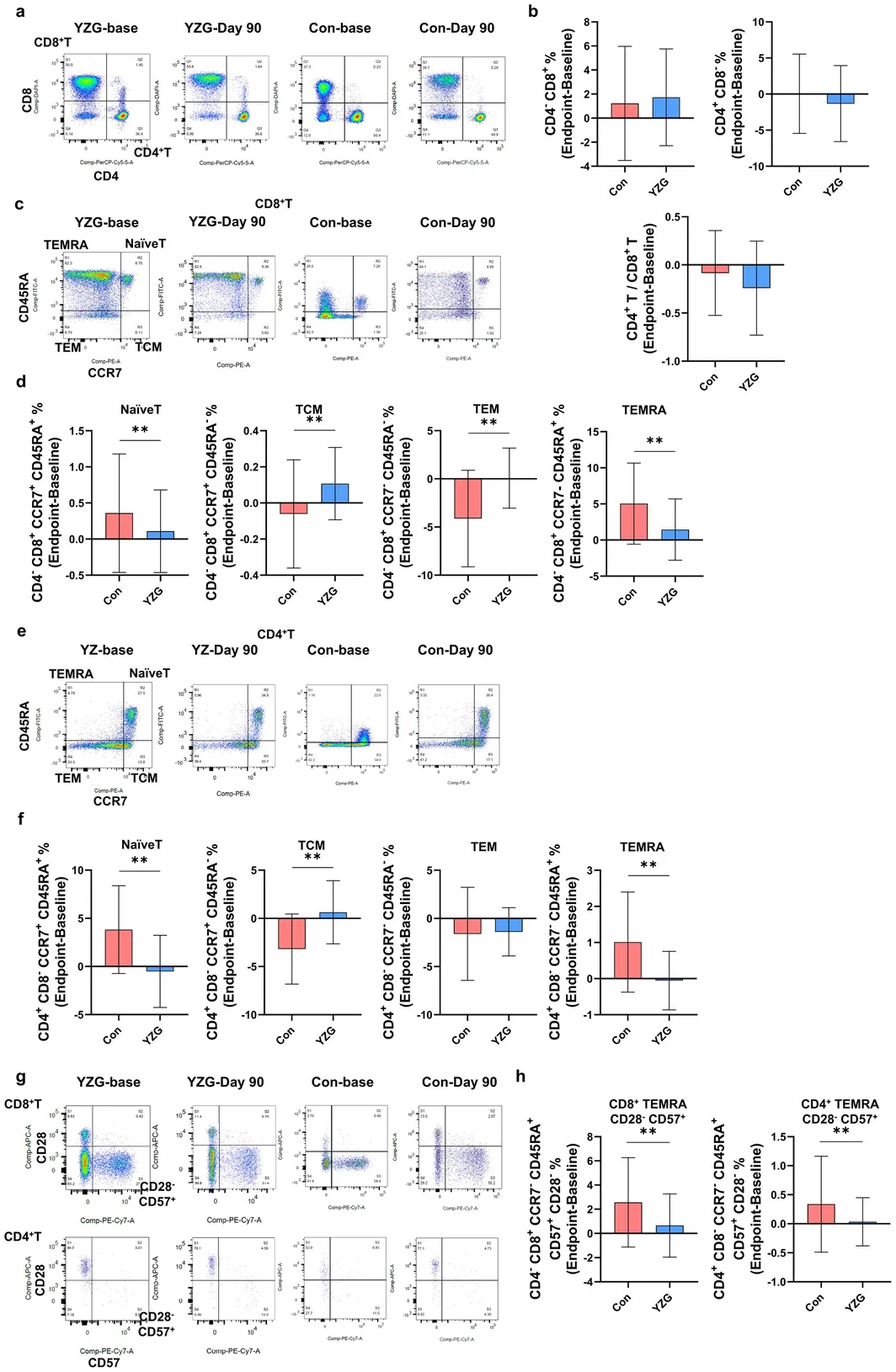
Effect of YZG on T cells in elderly population. (a, b) Changes in CD8^+^ T cells, CD4^+^ T cells and CD4^+^ T/CD8^+^ T ratio; (c–f) Changes of Naïve T cells, TCM cells, TEM cells and TEMRA cells of CD8^+^ and CD4^+^; (g, h) CD28^−^ CD57^+^ TEMRA cell changes of CD8^+^ and CD4^+^. It's the percentage of these cells in CD45^+^ cell population; **p < 0.01.

Flow cytometry was performed in the peripheral blood of the placebo group (Control) and the intervention group (YZG) before and after the intervention. The flow gate strategy and the situation of T cells between the Day 90 and baseline of the YZG and control groups (Figure S2). After 90 days of intervention, there were no significant differences in the percentage of CD8^+^ T cells and CD4^+^ T cells and the ratio of CD4^+^ T/CD8^+^ T between the YZG group and the control group. After 90 days of intervention, significant decreases in CD8^+^ TCM, CD4^+^ TCM, and CD8^+^ TEM were observed in the control group, while significant increases were noted in CD8^+^ TEMRA, CD4^+^ TEMRA, CD28^−^ CD57^+^ CD8^+^ TEMRA, and CD28^−^ CD57^+^ CD4^+^ TEMRA (*p* < 0.01). Following intervention with YZG, a significant increase in CD8^+^ TCM was observed, with no significant changes detected in the remaining subsets (*p* < 0.01) (Figures 4a,b and S3).

We compared the difference between the end point minus baseline in the YZG group and the end point minus baseline in the control group. The between‐group analysis in the YZG group showed a significantly lower increase in the percentage of Naïve CD8^+^ T cells and CD8^+^ TEMRA cells. The percentage of CD8^+^ TCM cells was significantly up‐regulated in the YZG group and down‐regulated in the control group after 90 days intervention. The percentage of CD8^+^ TEM cells was significantly down‐regulated in the control group, and no significant change was observed in the YZG group after 90 days intervention (Figure 4c,d). A similar trend was also observed in the CD4^+^ T cell subset (Figure 4e,f). In addition, the change in endpoints minus baseline differences between the two groups of these cell subsets was consistent with the change in the percentage of CD8^+^ T cells or CD4^+^ T cells in the CD45^+^ cell population (Figure S3b,c).

The CD28^−^ CD57^+^ TEMRA cells were significantly up‐regulated in the control group and no significant change in the YZG group after 90 days intervention (Figure 4g,h). The between‐group analysis in the YZG group showed a significantly lower increase in the percentage of CD28^−^ CD57^+^ TEMRA, compared with that in the control group. The trend of its variation remains consistent across different cell population proportions (Figure S3). Comparing the data of YZG group and control group, no significant results were observed in the percentage of DN (double negative) B cells (CD45^+^ CD3^−^ CD19^+^ IgD^−^ CD27^−^) and NK cells (CD45^+^ CD3^−^ CD56^+^) (Figure S4).

### 3.5 Yuzhu Gao Modulates Gut Microbiota Composition in the Elderly

**FIGURE 5:**
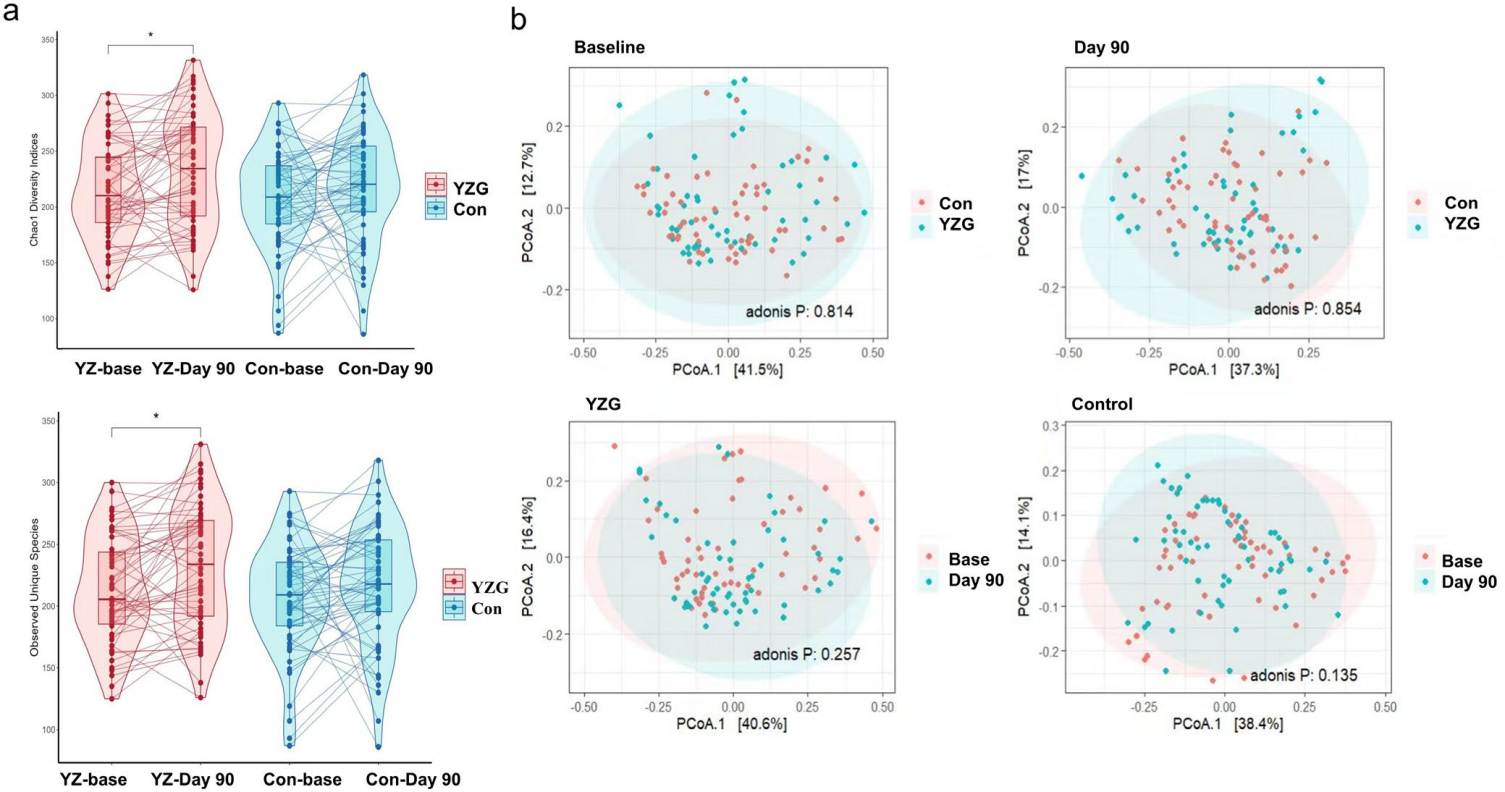
Effect of YZG on gut microbial diversity in elderly population. (a) Bacterial alpha diversity of baseline and endpoint participants in the YZG and Con groups; (b) Comparison of beta diversity of gut microbiota of baseline and endpoint participants in the YZG and Con groups; *p < 0.05.

In the alpha diversity analysis based on the obtained ASV feature sequences and ASV abundance tables, the Chao1 and observed species were significantly higher at Day 90 of the YZG group compared to that of the baseline, whereas there was no significant difference between the baseline and Day 90 of the control group (Figure 5a). The results demonstrated that no statistically significant differences in alpha diversity were observed either at baseline or after adjusting for relevant confounding factors (Table S3). The beta diversity among groups was characterized by weighted‐based PCoA analysis. There was no significant difference in the composition of microbial between the YZG and control groups (Figure 5b).

**FIGURE 6:**
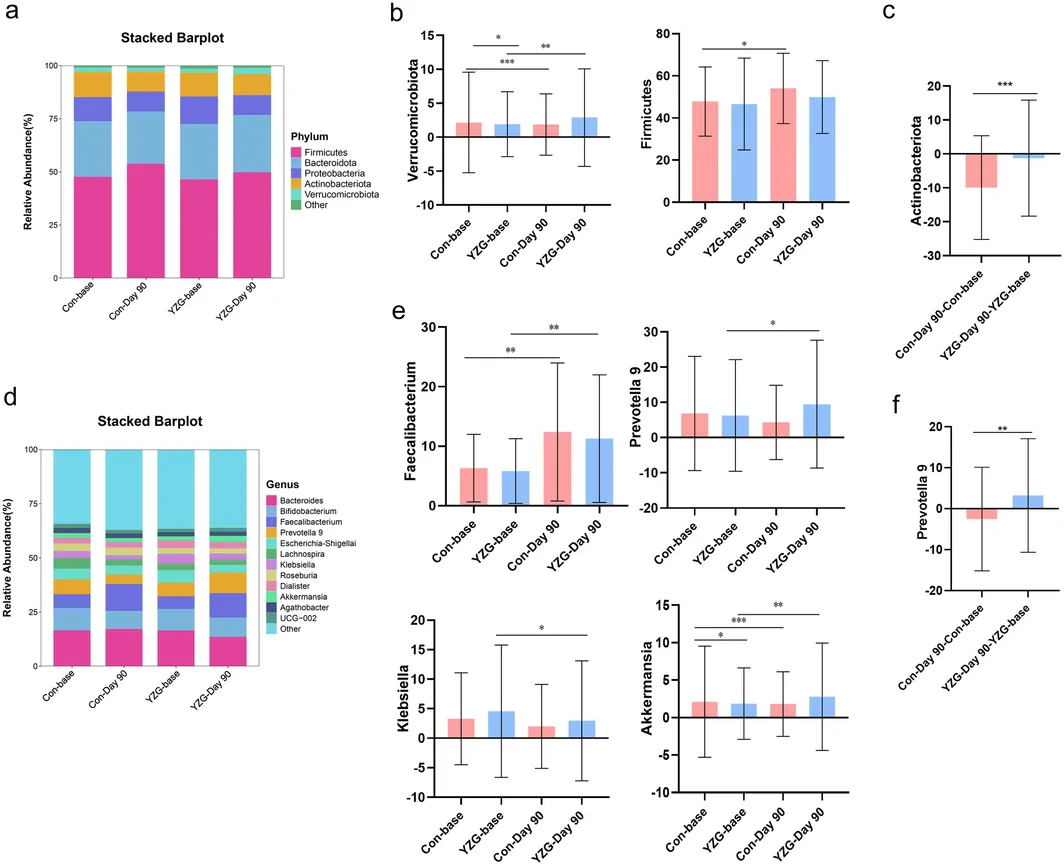
Composition of Gut Microbiota at Phylum and Genus Levels. (a, d) Composition of gut microbiota at the phylum and genus levels; (b, e) Differential bacterial among the TOP5 phyla and TOP12 genera in gut microbiota; (c, f) Differentially abundant bacterial in the TOP5 Phyla and TOP12 genera from baseline to day 90; *p < 0.05, **p < 0.01, ***p < 0.001.

The most relative abundance phylum was Firmicutes (50.2%), followed by *Bacteroidota* (25.7%), *Proteobacteria* (10.6%), and *Actinobacteriota* (10.2%). These four phyla accounted for about 96.9% of the gut microbiota. The composition of the gut microbiome of participants in the YZG and control groups was characterized at both baseline and Day 90 (Figure 6). Based on the analysis of species differences at the phylum level, compared to baseline, the relative abundance of *Firmicutes* increased in the control group on Day 90, while the relative abundance of *Verrucomicrobiota* decreased significantly and the relative abundance of *Bacteroidota* decreased. In the YZG group, the abundance of *Verrucomicrobiota* increased significantly. Evaluating the difference between Day 90 and baseline, the relative abundance of *Actinobacteriota* decreased more significantly in the control group compared to the YZG group (*p* < 0.001).

According to the analysis of abundance differences at the genus level, compared to baseline, the relative abundance of *Faecalibacterium* increased significantly in both the YZG and control groups on Day 90. The relative abundance of *Prevotella 9* and *Akkermansia* increased significantly in the YZG group, while there was no significant difference in the control group before and after intervention. The relative abundance of *Klebsiella* decreased significantly in the YZG group, while there was no significant difference in the control group before and after intervention. Evaluating the difference between Day 90 and baseline, the relative abundance of *Prevotella 9* was significantly higher in the YZG group compared to the control group.

**FIGURE 7:**
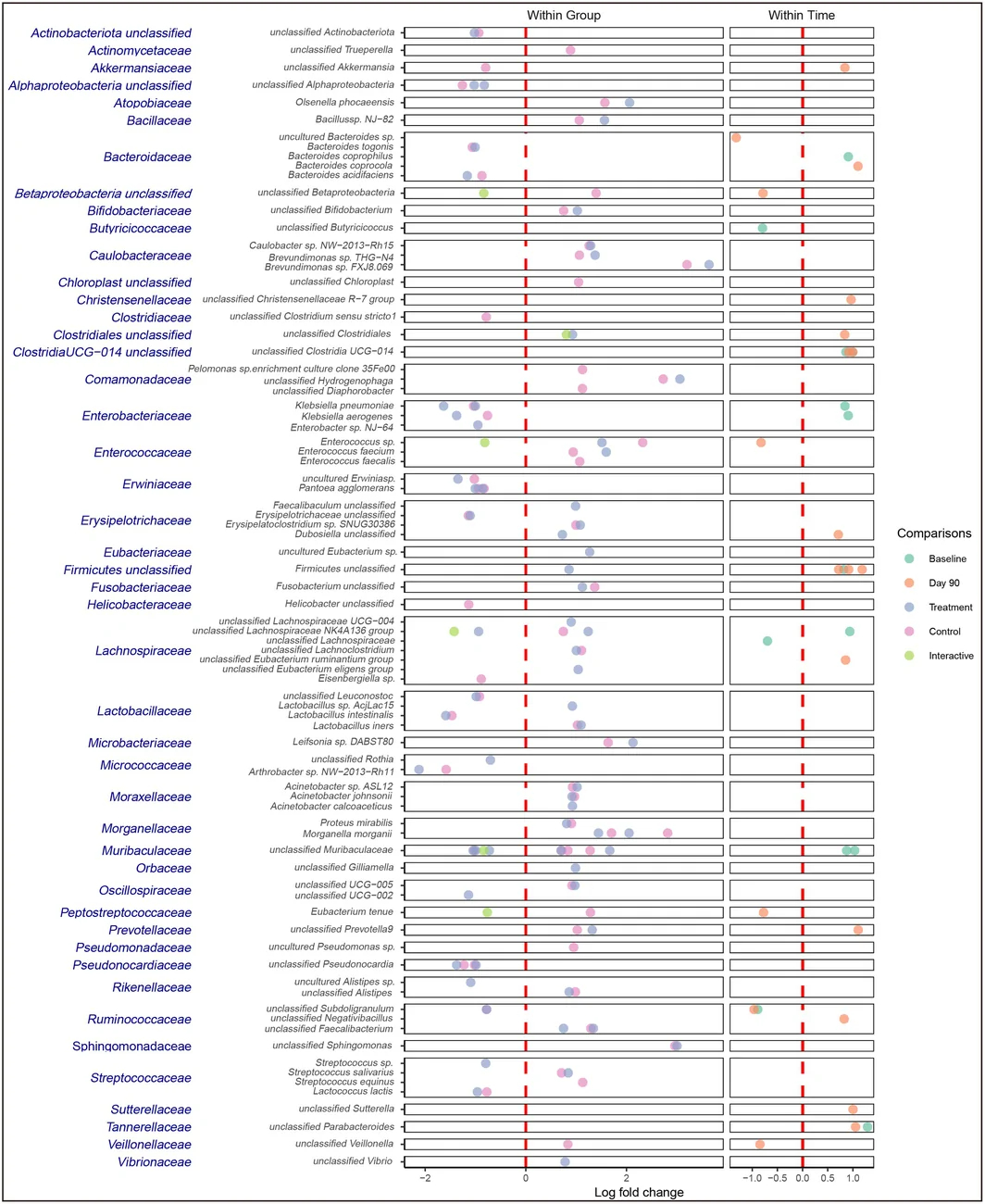
Differential bacteria among various elderly groups at the species‐level classification.

Based on the species‐level differences analysis (Figure 7, Table S4). We also conducted an analysis before and after intervention in the YZG group, and the key bacteria was figured out by cutting |logFC| ≥ 1.5. The relative abundance of *Brevundimonas* sp. *FXJ8.069*, *Enterococcus faecium* , *Morganella morganii, unclassified Hydrogenophaga*, *unclassified Sphingomonas*, *Olsenella phocaeensis*, *Enterococcus* sp., *Leifsonia* sp. *DABST80*, *unclassified Muribaculaceae*, and *Bacillus* sp. *NJ‐82* was significantly upregulated. Although the relative abundance of *Brevundimonas* sp. *FXJ8.069*, *unclassified Hydrogenophaga*, *Leifsonia* sp. *DABST80*, *unclassified Sphingomonas*, and *Olsenella phocaeensis* also increased significantly in the control group after intervention, this upward trend was more pronounced in the YZG group. Following intervention in the YZG group, the relative abundance of *Klebsiella pneumoniae* , *Lactobacillus intestinalis* , and *Arthrobacter* sp. *NW‐2013‐Rh11* was significantly downregulated (*p* < 0.01).

The intervention predominantly influences microbial composition by altering the relative abundance of species in the certain genera, including *Faecalibacterium, Prevotella 9*, *Akkermansia, Klebsiella, Enterococcus, Clostridiales, Bacteroides, Subdoligranulum, Sutterella, Eubacterium*, etc.

### 3.6 Yuzhu Gao Altered Immune Status Relates to the Alteration Gut Microbial Genus in the Elderly

**FIGURE 8:**
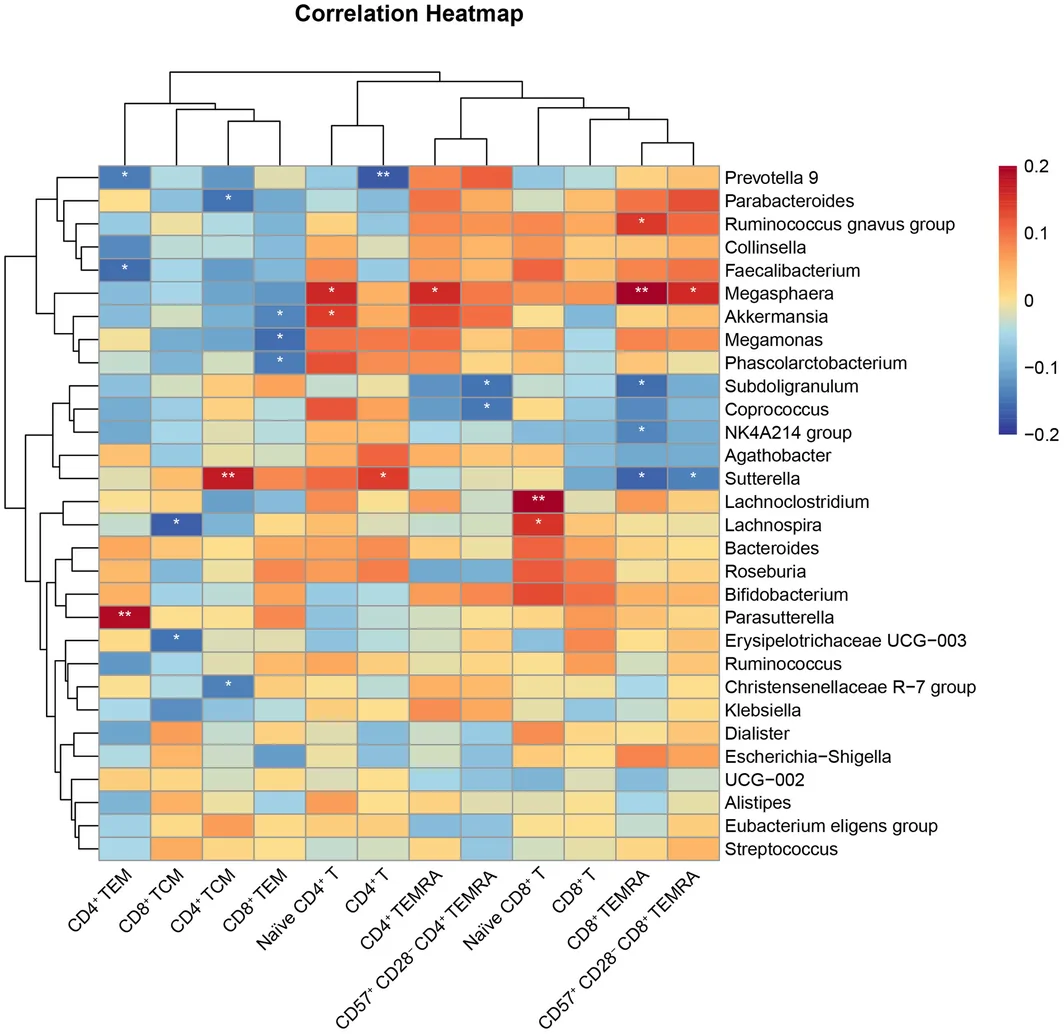
The relationship between microbiota and changes in immune parameters. *p < 0.05, **p < 0.01.

After the intervention, the correlation between T‐cell subsets and bacterial relative abundance at the genus level was analyzed using Spearman's correlation analysis, with the results presented in Figure 8. *Prevotella 9* was significantly negatively correlated with CD4^+^ T and CD4^+^ TEM (*r* = −0.143, *p* = 0.030); *Faecalibacterium* was significantly negatively correlated with CD4^+^ TEM (*r* = −0.155, *p* = 0.019); *Akkermansia* was significantly negatively correlated with CD8^+^ TEM (*r* = −0.133, *p* = 0.044) and positively correlated with naïve CD4^+^ T (*r* = 0.143, *p* = 0.030).

During the administration intervention period, the incidence of respiratory tract infections and the total symptom score were assessed through questionnaire follow‐up (Patel et al. 2026). Feedback was received from a total of 116 participants, with 56 in the intervention group (YZG) and 60 in the placebo group (Control). Compared with the control group, the incidence of respiratory tract infection in the YZG group was significantly decreased (Table 2).

**TABLE 2:** Incidence of respiratory infection.

Group	Examples	Not infected	Respiratory tract infection	*p*
YZG group	56	50	6	0.029*
Con group	60	44	16	

* Data analysis was performed using the chi‐square test.

We also analyzed the relationship between the respiratory tract infection (RTI) and immune and gut microbial index altered by Yuzhu Gao (YZG). The results demonstrated that the key bacterial genera (e.g., *Faecalibacterium, Prevotella 9*, *Akkermansia, Klebsiella, Subdoligranulum, Sutterella*) altered by YZG, correlating with enhanced immune function (particularly T‐cell profiles), showed a significantly impact of respiratory infections during the intervention period (Figures S5–S7).

## 4 Discussion

A dynamic bidirectional regulatory network exists between the gut microbiota and the host immune system (Ghosh and Sinha 2025; Wang et al. 2025). In this cohort study focusing on an elderly population, a significant association between gut microbiota composition and host immune cell subsets was investigated. The results showed that the relative abundances of key bacterial taxa, including *Bifidobacterium*, *Ruminococcaceae*, *Akkermansia*, and *Klebsiella*, exhibited specific correlations with various immune cell genealogies. Among these, *Akkermansia, Eubacterium* and *Parabacteroides* were significantly negatively correlated with TEMRA cells. There is a significant positive correlation between CD4^+^ TEMRA CD57^−^ CD28^+^ and CD4^+^ TEMRA cells and *Klebsiella*. The high abundance of *Akkermansia* is closely linked to positive health outcomes in various aging‐related diseases, and it is notably higher in the longevity group (Panzetta and Valdivia 2024). Additionally, *Prevotella* is associated with lower levels of depression and anxiety in patients with IBD and healthy individuals (Yuan et al. 2021). Members of the *Klebsiella* genus have rapidly evolved, resulting in multidrug‐resistant and highly virulent organisms closely related to infections in immunocompromised hosts, such as community‐acquired infections, posing a serious threat to global clinical and public health (Dong et al. 2022). *Akkermansia muciniphila* shows a significant positive correlation with the clinical response rate to PD‐1 immune checkpoint blockade therapy. The underlying mechanism may be closely related to the directed recruitment of CCR9^+^ CXCR3^+^ CD4^+^ T lymphocytes to the tumor microenvironment and the upregulation of interleukin‐12 (IL‐12) secretion levels (Routy et al. 2018). Another mechanistic study demonstrated that in Tfam^fl^/^fl^CD4^Cre^ conditional deletion mice, the abundance of beneficial commensal bacteria represented by *Lactobacillus* and *Ruminococcaceae* was significantly reduced (Gómez de Las Heras et al. 2025). This finding further untangles, from a genetic perspective, the reverse regulatory role of immune cell functional status on gut microbiota structure, corroborating the complex and precise interaction between the gut microbiota and the immune system. In summary, the dynamic interactive dialog between the gut microbiota and the host immune system holds significant biological importance in maintaining host‐microbiota symbiotic homeostasis and regulating the process of immunosenescence. This provides a crucial theoretical basis for understanding aging‐related immune function decline and for developing gut microecological intervention strategies.

There is a lack of clinical evidence regarding the impact of *P. odoratum* on immune parameters and gut microbiota. Our study demonstrates that YZG not only modulates gut microbial diversity by increasing the abundance of *Akkermansia* and *Prevotella 9* while reducing the abundance of *Klebsiella* and *Ruminococcus gnavus* groups, but also significantly decreases the percentage of CD4^+^ TEMRA, CD8^+^ TEMRA, and the more senescent CD28^−^ CD57^+^ CD4^+^ TEMRA and CD28^−^ CD57^+^ CD8^+^ TEMRA cells, thereby effectively enhancing the clearance efficiency of senescent immune cells in the peripheral blood of the elderly. Concurrently, this study observed that YZG significantly reduces the risk of respiratory tract infections in the elderly population. Comparative analysis of gut microbiota structure and T‐cell phenotypes between elderly subjects with and without respiratory tract infections further confirmed the synergistic regulatory role of gut microbiota and T‐cell phenotypes in delaying disease progression in the elderly. Respiratory tract infection represents a prevalent health concern in the elderly population, especially during winter, and is closely linked to age‐associated alterations in immune indicators (Sjögren et al. 2008). As a traditional Chinese medicinal herb, *P. odoratum* is widely used in the YZG of respiratory tract infections due to its properties of nourishing yin and moistening the lungs. We collected information on the incidence rate of colds to observe the effect of improvement and analyzed the relationship among infection, immune cells, and gut microbiota. The study found that the trend of changes in immune cells was consistent whether infection occurred (Figure S6). Furthermore, previous clinical studies (Chen et al. 2024) have primarily focused on age‐related changes in T cells among the elderly, while research and exploration into traditional Chinese medicine or botanical drugs targeting TEMRA cells to delay immunosenescence remain insufficient.

The potential mechanism underlying the pharmacological effects of YZG may involve modulating gut microbiota structure, promoting the proliferation of beneficial bacteria, and inducing the production of various beneficial metabolites, thereby mediating immunoregulation (Ghosh and Sinha 2025). Simultaneously, it may also be closely associated with improving T‐cell immune defense function and inhibiting the colonization of harmful gut bacteria. Previous studies have confirmed that *Akkermansia muciniphila* is significantly enriched in the gut microbiota of long‐lived elderly individuals. This bacterium can synthesize and elevate beneficial metabolites such as short‐chain fatty acids, which regulate T‐cell differentiation by inhibiting the release of pro‐inflammatory cytokines and mitigating chronic inflammatory responses and oxidative stress damage (Alexander et al. 2024; Shaheen et al. 2025). Another study indicated that supplementation with *Akkermansia muciniphila* preparations can promote the infiltration of CAR‐T cells into bone marrow tissue, reverse the imbalance in the CD4/CD8 CAR‐T cell ratio, induce Tc1‐type CD8^+^ T cell polarization, and promote the release of tryptophan‐derived indole metabolites, ultimately enhancing the body's immune surveillance and control over tumors (Marcos‐Kovandzic et al. 2025). As core effector cells of the adaptive immune system, the normalization of T‐cell function can effectively mediate pathogen clearance, tissue damage repair, and establish long‐term immune memory against respiratory bacterial infections. Research has confirmed that the B and T lymphocyte attenuator (BTLA) can mediate CD4^+^ T cell exhaustion by inhibiting T‐cell activation, proliferation, and cytokine secretion, or by inducing CD4^+^ T cell apoptosis. Intervention with neutralizing anti‐BTLA antibodies significantly reduces the colonization load of *Klebsiella pneumoniae* and lowers mortality in mouse models of hepatitis B virus‐associated acute‐on‐chronic liver failure (Yu et al. 2024). In summary, the results of this study fully corroborate the unique pharmacological advantage of YZG as a traditional Chinese medicine for multi‐target and multi‐pathway intervention, suggesting broad application prospects for YZG in the prevention of infectious diseases and the control of chronic non‐communicable diseases in the elderly population.

The present study has some strengths and weaknesses. First, this is the first randomized clinical trial that investigated the correlations between immune cell subsets and gut microbiota abundance in the elderly cohort recruited from the Tianjin community, and the effect of *P. odoratum* supplementation on elderly individuals, especially on respiratory tract infection, T cell subsets and gut microbiota homeostasis. Additionally, individuals had high adherence to treatment considering regular weekly reminders and follow‐up. However, it limits the generalizability of our findings to the Tianjin elderly community population. Several covariates such as blood index, weight, age, and gender was considered in this trial; nevertheless, other confounders such as the presence of comorbid viral infectious diseases such as cytomegalovirus infection in the elderly population which may affect our results remained to be assessed. Furthermore, evaluations of respiratory infection symptoms of participants were based on self‐reported data which might be subject to various biases such as reactivity or selective‐reporting bias. Importantly, no functional immune assays (e.g., anti‐viral T cell responses) were performed in this study. Thus, the observed correlations between YZG treatment, immune cell subsets, and self‐reported cold episodes do not establish causation. Functional studies are needed to confirm anti‐viral immune effects. Finally, While we characterized gut microbial composition at the bacterial level using 16S rRNA sequencing, we did not measure microbial metabolites or structural components (e.g., short‐chain fatty acids, secondary bile acids, tryptophan metabolites, lipopolysaccharide) that could directly influence T‐cell differentiation, activation, or senescence. These functional outputs are critical mediators of host‐microbe communication. In summary, *P. odoratum*, a food homology and medicinal herb, commonly, can enhance the immune function, and assist in the treatment of various chronic diseases. This clinical study revealed that *P. odoratum* is suitable for the elderly population daily supplementation, can improve immunity, alleviate aging‐related intestinal dysfunction, and prevent respiratory infections.

## Author Contributions

All authors participated in the conceptualization and design of this study. Tingting Xia, Tianyi Cui, and Xin Zhao were responsible for data analysis, organization, and initial manuscript drafting. Kaijun Niu and Hongmei Wu established the clinical cohort, managed clinical trial registration, and collected clinical samples and information. Wenzhi Yang and Min Zhang performed the chemical characterization of YZG samples. Tingting Xia, Tianyi Cui, Jiayao An, Zhi Cui, Xinqin Zhong, and Bin Lv assisted in sample processing and testing. Yuhong Huang and Shengyuan Zhou facilitated ethical registration. Yongsheng Wu and Donglan Yan provided *P. odoratum * (YZG) samples. Fan Jia and Lin Zhang contributed to data analysis and visualization. Xin Zhao and Xiumei Gao oversaw manuscript revision and project supervision. All authors have reviewed and approved the final version of the manuscript.

## Funding

This work was supported by the National Key R&D Program of China [2022YFC3500300, 2022YFC3500305], and the Science & Technology Development Fund of Tianjin Education Commission for Higher Education [2022ZD039].

## Ethics Statement

Ethical approval was given by the Research Ethics Committee of the Second Affiliated Hospital of Tianjin University of Chinese Medicine (Project No. EC.AT/03.19‐01/10.0) and was conducted in accordance with the guidelines set out in the Declaration of Helsinki. All the participants gave written informed consent.

## Consent

The authors have nothing to report.

## Conflicts of Interest

The authors declare no conflicts of interest.

## Supporting information


**Figure S1:** Base peak chromatograms of mixed standards and YZG in positive and negative ion modes. (a) Base Peak Chromatogram in Negative Ion Mode; (b) Base Peak Chromatogram in positive Ion Mode.
**Figure S2:** Gating strategy for T cell subsets analyzed by fiow cytometry.
**Figure S3:** Effect of YZG on T cells in elderly population. (a) CD8^+^ T cells and CD4^+^ T cells in each group between the Day 90 and the baseline; (b), (c) The changes of Naïve T cells, TCM cells, TEM cells, and TEMRA cells of CD8^+^and CD4^+^, as the percentage of this cell population in CD8^+^ T cells and CD4^+^ T cells; (d), (e) The situation between the Day 90 and baseline of CD8^+^ and CD4^+^ Naïve T cells, TCM cells, TEM cells, TEMRA cells in each group; (f) The changes of CD28^−^CD57^+^ TEMRA cells of CD8^+^and CD4^+^ is the percentage of this cell population in CD8^+^ and CD4^+^ TEMRA cells; (g) The situation between the Day 90 of CD8^+^and CD4^+^CD28^−^CD57^+^ TEMRA cells in each group and the baseline; *p < 0.05, **p < 0.01.
**Figure S4:** Effects of YZG on B cells and NK cells in elderly population. (a) Gating strategy for flow cytometry of B cells and NK cells; (b) Effects of YZG on DN B cells (CD27^−^IgD^−^); (c) Effects of YZG on NK cells; *p < 0.05, **p < 0.01.
**Figure S5:** Alteration of the other T cells in respiratory infections group during intervention. (a) Changes in CD8^+^ T cells, CD4^+^ T cells, and the CD4^+^/CD8^+^ T cell ratio; (b), (c) Changes in Naïve T cells, TCM cells, and TEM cells among both CD8^+^ and CD4^+^ populations; *p < 0.05, **p < 0.01, ***p < 0.001.
**Figure S6:** Alteration of the key T cells in respiratory infections group during intervention. (a) Changes of TEMRA cells of CD8^+^ and CD4^+^; (b) Changes of CD28^−^ CD57^+^ TEMRA cells of CD8^+^ and CD4^+^; *p < 0.05, **p < 0.01.
**Figure S7:** Alteration of the key gut microbiota in respiratory infections' group during intervention. (a) Changes in gut microbiota at the genus level in the YZG group with and without respiratory infections; (b) Changes in gut microbiota at the genus level in the Con group with and without respiratory infections; *p < 0.05, **p < 0.01, ***p < 0.001.
**Table S1:** Identification list of components in the YZG.
**Table S2:** Immune cells and antibody markers.
**Table S3:** Hematological results of the YZG intervention group and the placebo group in the elderly population.
**Table S4:** Statistics analysis of bacterial alpha diversity among groups.

## Data Availability

The 16S rRNA gene sequencing data generated in this study have been deposited in the National Center for Biotechnology Information (NCBI) Sequence Read Archive under the accession numbers PRJNA1203376. In compliance with ethical requirements for human subject research, other data will be shared upon formal request from the corresponding authors with appropriate institutional approvals and data protection agreements in place.

## References

[acel70724-bib-0001] Alexander, M. , V. Upadhyay , R. Rock , et al. 2024. “A Diet‐Dependent Host Metabolite Shapes the Gut Microbiota to Protect From Autoimmunity.” Cell Reports 43, no. 11: 114891. 10.1016/j.celrep.2024.114891. PMC11660937 39500329

[acel70724-bib-0002] Chen, Y. , X. Li , M. Yang , et al. 2024. “Time‐Restricted Eating Reveals a ‘Younger’ Immune System and Reshapes the Intestinal Microbiome in Human.” Redox Biology 78: 103422. 10.1016/j.redox.2024.103422. PMC11616606 39561680

[acel70724-bib-0003] Colica, C. , and I. Vecchio . 2026. “Gut Microbiota: Origin or Panacea for All Ills? Immune and Metabolic Diseases, Nutrition, and Microbiota‐Based Interventions.” Microbial Pathogenesis 212: 108213. 10.1016/j.micpath.2025.108213. 41317880

[acel70724-bib-0004] Ding, S. Q. , X. Y. Lyu , S. Y. Zhou , et al. 2025. “Metformin‐Associated Gut Microbiota Remodeling Correlates With Reinvigorated Splenic Immunity in Aged Mice: Microbiome‐Immune Crosstalk via the Gut‐Spleen Axis.” Frontiers in Immunology 16: 1633486. 10.3389/fimmu.2025.1633486. PMC12507646 41080580

[acel70724-bib-0005] Dong, N. , X. Yang , E. W. Chan , R. Zhang , and S. Chen . 2022. “Klebsiella Species: Taxonomy, Hypervirulence and Multidrug Resistance.” eBioMedicine 79: 103998. 10.1016/j.ebiom.2022.103998. PMC9010751 35405387

[acel70724-bib-0006] Ghosh, N. , and K. Sinha . 2025. “Guardians Within: Cross‐Talk Between the Gut Microbiome and Host Immune System.” World Journal of Gastrointestinal Pathophysiology 16, no. 4: 111245. 10.4291/wjgp.v16.i4.111245. PMC12754317 41479870

[acel70724-bib-0007] Gómez de Las Heras, M. M. , E. Carrasco , M. Pérez‐Manrique , et al. 2025. “CD4 T Cell Therapy Counteracts Inflammaging and Senescence by Preserving Gut Barrier Integrity.” Science Immunology 10, no. 110: eadv0985. 10.1126/sciimmunol.adv0985. PMC7618201 40749035

[acel70724-bib-0008] Gu, P. , R. Wei , R. Liu , et al. 2025. “Aging‐Induced Alternation in the Gut Microbiota Impairs Host Antibacterial Defense.” Advanced Science (Weinheim) 12, no. 12: e2411008. 10.1002/advs.202411008. PMC11948050 39792643

[acel70724-bib-0009] Lance, A. J. , S. Kai , V. P. Akhil , et al. 2026. “Clonal Expansion of Cytotoxic CD8^+^ T Cells in Lecanemab‐Associated ARIA.” Nature Communications 17, no. 1: 2180. 10.1038/s41467-026-68921-3. PMC12960807 41617706

[acel70724-bib-0010] Liang, H. , X. Ding , S. Liu , et al. 2026. “Aging‐Caused the Changes of the Gut Microbiota Drive Intestinal Barrier Dysfunction and Increase Sepsis Susceptibility.” Gut Microbes 18, no. 1: 2630475. 10.1080/19490976.2026.2630475. PMC12928652 41723572

[acel70724-bib-0011] Liu, J. R. , B. X. Chen , J. Q. Huang , et al. 2024. “Fingerprinting and Characterization of the Polysaccharides From Polygonatum Odoratum and the In Vitro Fermented Effects on *Lactobacillus johnsonii* .” Journal of Pharmaceutical and Biomedical Analysis 239: 115911. 10.1016/j.jpba.2023.115911. 38091818

[acel70724-bib-0012] Marcos‐Kovandzic, L. , M. Avagliano , M. Ben Khelil , et al. 2025. “Gut Microbiota Modulation Through Akkermansia spp. Supplementation Increases CAR T‐Cell Potency.” Cancer Discovery 15, no. 9: 1905–1926. 10.1158/2159-8290.Cd-24-1230. PMC12409280 40498998

[acel70724-bib-0013] Nan, Y. , H. Liang , X. Pang , et al. 2023. “Qualitative and Quantitative Studies on Two Commercial Specifications of Polygonatum Odoratum.” Frontiers in Chemistry 11: 1146153. 10.3389/fchem.2023.1146153. PMC9995655 36909715

[acel70724-bib-0014] Panzetta, M. E. , and R. H. Valdivia . 2024. “Akkermansia in the Gastrointestinal Tract as a Modifier of Human Health.” Gut Microbes 16, no. 1: 2406379. 10.1080/19490976.2024.2406379. PMC11418289 39305271

[acel70724-bib-0015] Patel, M. I. , M. Voskanyan , H. Agajanian , R. Agajanian , Y. Podnos , and A. Milstein . 2026. “A Lay Health Worker‐Led Symptom Intervention and Acute Care Use in Older Adults With Cancer: A Randomized Clinical Trial.” JAMA 335, no. 8: 674–681. 10.1001/jama.2025.23403. PMC12754701 41468027

[acel70724-bib-0016] Routy, B. , E. Le Chatelier , L. Derosa , et al. 2018. “Gut Microbiome Influences Efficacy of PD‐1‐Based Immunotherapy Against Epithelial Tumors.” Science 359, no. 6371: 91–97. 10.1126/science.aan3706. 29097494

[acel70724-bib-0017] Salumets, A. , L. Tserel , A. P. Rumm , et al. 2022. “Epigenetic Quantification of Immunosenescent CD8(+) TEMRA Cells in Human Blood.” Aging Cell 21, no. 5: e13607. 10.1111/acel.13607. PMC9124311 35397197

[acel70724-bib-0018] Schoultz, I. , M. J. Claesson , M. G. Dominguez‐Bello , et al. 2025. “Gut Microbiota Development Across the Lifespan: Disease Links and Health‐Promoting Interventions.” Journal of Internal Medicine 297, no. 6: 560–583. 10.1111/joim.20089. PMC12087861 40270478

[acel70724-bib-0019] Shaheen, N. , W. Khursheed , B. Gurung , and S. Wang . 2025. “ *Akkermansia muciniphila* : A Key Player in Gut Microbiota‐Based Disease Modulation.” Microbiological Research 301: 128317. 10.1016/j.micres.2025.128317. 40845731

[acel70724-bib-0020] Sjögren, P. , E. Nilsson , M. Forsell , O. Johansson , and J. Hoogstraate . 2008. “A Systematic Review of the Preventive Effect of Oral Hygiene on Pneumonia and Respiratory Tract Infection in Elderly People in Hospitals and Nursing Homes: Effect Estimates and Methodological Quality of Randomized Controlled Trials.” Journal of the American Geriatrics Society 56, no. 11: 2124–2130. 10.1111/j.1532-5415.2008.01926.x. 18795989

[acel70724-bib-0021] Wang, Q. , Q. Meng , Y. Chen , et al. 2025. “Interaction Between Gut Microbiota and Immunity in Health and Intestinal Disease.” Frontiers in Immunology 16: 1673852. 10.3389/fimmu.2025.1673852. PMC12641010 41293162

[acel70724-bib-0022] Wang, Y. , C. Dong , Y. Han , Z. Gu , and C. Sun . 2022. “Immunosenescence, Aging and Successful Aging.” Frontiers in Immunology 13: 942796. 10.3389/fimmu.2022.942796. PMC9379926 35983061

[acel70724-bib-0023] Yu, X. , F. Yang , Z. Shen , et al. 2024. “BTLA Contributes to Acute‐on‐Chronic Liver Failure Infection and Mortality Through CD4(+) T‐Cell Exhaustion.” Nature Communications 15, no. 1: 1835. 10.1038/s41467-024-46047-8. PMC10901893 38418488

[acel70724-bib-0024] Yuan, X. , B. Chen , Z. Duan , et al. 2021. “Depression and Anxiety in Patients With Active Ulcerative Colitis: Crosstalk of Gut Microbiota, Metabolomics and Proteomics.” Gut Microbes 13, no. 1: 1987779. 10.1080/19490976.2021.1987779. PMC8632339 34806521

[acel70724-bib-0025] Zhao, P. , C. Zhao , X. Li , et al. 2018. “The Genus Polygonatum: A Review of Ethnopharmacology, Phytochemistry and Pharmacology.” Journal of Ethnopharmacology 214: 274–291. 10.1016/j.jep.2017.12.006. 29246502

[acel70724-bib-0026] Zhao, P. , H. Zhou , C. Zhao , et al. 2019. “Purification, Characterization and Immunomodulatory Activity of Fructans From Polygonatum Odoratum and P. Cyrtonema.” Carbohydrate Polymers 214: 44–52. 10.1016/j.carbpol.2019.03.014. 30926006

[acel70724-bib-0027] Zhu, X. , X. Huang , M. Hu , et al. 2024. “A Specific Enterotype Derived From Gut Microbiome of Older Individuals Enables Favorable Responses to Immune Checkpoint Blockade Therapy.” Cell Host & Microbe 32, no. 4: 489–505.e5. 10.1016/j.chom.2024.03.002. 38513657

