# Supplementary material for: Associations Between Gut Microbiota and Immune Profiles in Community‐Dwelling Older Adults and the Regulatory Role of Polygonatum odoratum
Source: Aging Cell. 2026 Sep 23;25(10):e70724. doi: 10.1111/acel.70724 (PMC13599344; doi:10.1111/acel.70724)
Supplement: Supplementary file 1 [file ACEL-25-e70724-s001.doc]

**Additional information**

**Supplementary material and methods**

***Flow cytometry typing and quality control***

Data acquisition was performed using a BD FACSAria III flow cytometer. Prior to each experiment, quality control was conducted according to the instrument's daily quality control procedure using CST beads to confirm stable instrument performance before proceeding with sample analysis. The compensation matrix was generated automatically by the system's compensation program based on appropriate single-stained controls and compensation beads. Gating for each cell population was established in combination with unstained controls, single-positive controls, and/or fluorescence minus one (FMO) control to ensure the accuracy of positive population identification and the reliability of the results.

Peripheral blood in EDTA anticoagulated tubes, was subjected to erythrocyte lysis twice, then centrifuged at 4°C 350×g for 10 min to obtain the precipitate, which was fully resuspended by adding 1 mL of PBS buffer containing 2% serum (2% PB buffer). Fc receptor blockers (422302, Human TruStain FcX ^TM^, Biolegend) and flow antibodies were added sequentially: CD45, CD3, CD4, CD8, CCR7, CD45RA, CD28, CD57 (368526, Brilliant Violet 510^TM^ anti-human CD45; 300470, APC/Fire750^TM^ anti-human CD3; 300530, PerCP/Cyanine5.5 anti-human CD4; 301036, Brilliant Violet 421 ^TM^ anti-human CD8a; 353204, PE anti-human CD197 (CCR7); 304106, FITC anti-human CD45RA; 302912, APC anti-human CD28; 359624, PE/Cyanine7 anti-human CD57; 368526, Brilliant 302206, FITC anti-human; Biolegend). Incubate at 37℃ for 30 min away from light, centrifuge at 400×g at 4℃ for 5 min, discard the supernatant; 1mL of 2% PB buffer was added for suspension and prepared for use at 4℃ under dark light (BD FACS Aria flow cytometry, BD Biosciences).

***16S rRNA gene sequencing and microbial analysis***

Fresh fecal samples were collected under sterile conditions and immediately frozen at −80°C. Genomic DNA was extracted and assayed for concentration in accordance with previous studies(Zhang et al., 2023). The V3-V4 regions of the 16S rRNA gene were amplified with primers 341F:5’-CCTACGGGNGGCWGCAG-3’ and 805R:5’-GACTACHVGGGTATCTAATCC-3’, purified and quantified according to the manufacturer’s protocol. Purified amplicons were then paired-end sequenced on an Illumina NovaSeq 6000 (PE250), provided by LC-Bio Technology Co., Ltd., Hangzhou, China. The raw data was deposited in NCBI Sequence Read Archive with accession number PRJNA1203376. Raw reads were quality-filtered and merged by FLASH, and the taxonomy assignment was analysed according to previous study(Zhang et al., 2024),

***Other variables***

Before the intervention, sociodemographic data (age, gender, years of education, lifestyle habits, disease history were obtained through a standardized structured questionnaire, body mass index [BMI] were measured by a body composition analyzer (InBody120, InBody) and a tape measure. After 10 min of centrifuge at 3500 rpm, serum was separated from the peripheral blood and kept at −80 °C for future assessments. Hematology routine tests, including blood routine tests (6800Put, Mindray), liver and kidney function and lipid tests (UA5800, Beckman), are provided by Tianjin Dean Medical Laboratory Co., LTD. All samples were delivered and processed within 3 hours after venipuncture on the same day.

***Statistical analysis***

Statistical analysis was performed by Graphpad 8.0.1 software. Qualitative data were represented by percentage (%), and Chi-square test was applied for analysis. If the quantitative data obeyed a normal distribution, they were represented by Mean±SD. The changes in indicators within each intervention group before and after the intervention, were calculated by the differences between the two timepoints (the value at 90 days minus the value at baseline). One-way ANOVA test was used for multi-group comparison, and unpaired t-test was used for comparison between two groups. If the data does not follow normal distribution, the median and inter quantile range is used, and the Kruskal-Wallis test is used for multi-group comparison. The two groups comparison was performed by a Mann-Whitney test. Two sided tests were used in all statistical analyses, and *P* < 0.05 was considered statistically significant.

The 16S rRNA sequencing data were analyzed using R-4.4.2 and Graphpad 8.0.1 software. For alpha diversity analysis, violin plots were constructed using the ggplot2 (v3.5.0) extension package (ggsignif v0.6.4, ggrepel v0.9.5, ggpubr v0.6.0) in R statistical software. Group comparisons were performed using the Wilcoxon rank-sum test (two-sided). A multiple linear regression model (MLR) was established to quantify the effects of YZG factors, focusing on the regression coefficient of the interaction term between YZG and time, as well as its 95% confidence interval (95% CI). Beta diversity analysis was conducted using the phyloseq package (v1.48.0): Principal Coordinates Analysis (PCoA) was performed based on the Bray-Curtis distance matrix, and Permutational Multivariate Analysis of Variance (PERMANOVA, adonis2 function) was used to evaluate differences between groups, with 1000 permutation tests performed. For multi-group comparisons of species abundance at the phylum and genus levels, the Kruskal-Wallis test was used, with differences calculated as Day 90 minus Baseline (Day 0) values. Differences in species abundance at the species level were compared using the Analysis of Composition of Microbiomes with Bias Correction (ANCOM-BC) method(Lin & Peddada, 2020). The correlation between microbiota and immunity was evaluated using Spearman's rank correlation coefficient. Statistical significance was indicated as *P* < 0.05.

**Supplementary results:**

***Supplementary Figure:***

**Supplementary Figure S1**: Base peak chromatograms of mixed standards and YZG in positive and negative ion modes. (a) Base Peak Chromatogram in Negative Ion Mode; (b) Base Peak Chromatogram in positive Ion Mode.

**Supplementary Figure S2**: T cell flow cytometry ring-gate strategy.

**Supplementary Figure S3**: Effect of YZG on T cells in elderly population. (a) CD8^+^T cells and CD4^+^T cells in each group between the Day 90 and the baseline; (b), (c) The changes of Naïve T cells, TCM cells, TEM cells, and TEMRA cells of CD8^+^and CD4^+^, as the percentage of this cell population in CD8^+^T cells and CD4^+^T cells; (d), (e) the situation between the Day 90 and baseline of CD8^+^T and CD4^+^Naïve T cells, TCM cells, TEM cells, TEMRA cells in each group; (f) The change of CD28^-^CD57^+^TEMRA cells of CD8^+^and CD4^+^ is the percentage of this cell population in CD8^+^ and CD4^+^TEMRA cells; (g) The situation between the Day 90 of CD8^+^and CD4^+^CD28^-^CD57^+^TEMRA cells in each group and the baseline; *^*^P* < 0.05, *^**^P* < 0.01.

**B**

**C**

**CD19**

**CD19^+^**

**CD45^+^**

**CD3^-^**

**IgD**

**CD27**

**CD27^-^ IgD^-^**

**DN B cell**

**B cell**

**Pol-base**

**Pol-end**

**Con-base**

**Con-end**

**NK cell**

**CD56**

**CD16**

**CD56^+^**

**CD16^+^**

**CD56^+^**

**CD16^-^**

**Pol-base**

**Pol-end**

**Con-base**

**Con-end**

**Supplementary Figure S4**: Effects of YZG on B cells and NK cells in elderly population. (a) Gating strategy for flow cytometry of B cells and NK cells; (b) Effects of YZG on DN B cells (CD27^-^IgD^-^); (c) Effects of YZG on NK cells; *^*^P* < 0.05, *^**^P* < 0.01.

**Supplementary Figure S5**: Alteration of the other T cells in respiratory infections group during intervention. (a) Changes in CD8^+^ T cells, CD4^+^ T cells, and the CD4^+^/CD8^+^ T cell ratio; (b), (c) Changes in Naïve T cells, TCM cells, and TEM cells among both CD8^+^ and CD4^+^ populations; *^*^P* < 0.05, *^**^P* < 0.01, *^***^P* < 0.001.

**Supplementary Figure S6**: Alteration of the key T cells in respiratory infections group during intervention. (a) Changes of TEMRA cells of CD8^+^ and CD4^+^; (b) Changes of CD28^-^ CD57^+^ TEMRA cells of CD8^+^ and CD4^+^; *^*^ P＜0.05,^**^P*＜0.01.

**Supplementary Figure S7**: Alteration of the key gut microbiota in respiratory infections’ group during intervention. (a) Changes in gut microbiota at the genus level in the YZG group with and without respiratory infections; (b) Changes in gut microbiota at the genus level in the Con group with and without respiratory infections; *^*^ P*＜0.05*,^**^P*＜0.01*,^***^P*＜0.001.

***Supplementary Table：***

**Table S1**. Identification list of components in the YZG

| **NO.** | **t_R_**  **(min)** | **Observed**  ***m/z*** | **Adducts** | **Formula** | **Error**  **(ppm)** | **MS/MS** | **Identification** |
| --- | --- | --- | --- | --- | --- | --- | --- |
| 1 | 6.49 | 609.1470 | [M-H]^-^ | C_27_H_30_O_16_ | 1.46 | 300.0276,271.0230,255.0287 | rutin |
| 2 | 6.74 | 298.1076 | [M-H]^-^ | C_17_H_17_NO_4_ | -2.95 | 280.0967,160.0401 | *N*-*trans*-*p*-coumaroyloctopamine/isomer |
| 3 | 7.37 | 312.1228 | [M+H]^+^ | C_18_H_17_NO_4_ | -0.96 | 177.0542 | actinodaphnine/isomer |
| 4 | 7.38 | 328.1184 | [M-H]^-^ | C_18_H_19_NO_5_ | -1.96 | 310.1076,161.0237 | *N*-*cis*-feruloyloctopamine/isomer |
| 5 | 9.52 | 1241.5773 | [M-H_2_O+H]^+^ | C_57_H_94_O_30_ | -1.91 | 755.4229,575.3573,413.3050,395.2951 | 26-*O*-(*β*-*D*-glucopyranosyl)-furost-5-ene-3*β*,14*α*,22*α*,26-tetraol 3-*O*-*β*-*D*-glucopyranosyl-(1→2)-[*β*-*D*-glucopyranosyl-(1→3)]-*β*-*D*-glucopyranosyl-(1→4)-*β*-*D*-galactopyranoside/isomer |
| 6 | 9.90 | 1211.5663 | [M-H_2_O+H]^+^ | C_56_H_92_O_29_ | -2.28 | 1251.5629,1211.5662,755.4208,593.3687,575.3580,557.3459,413.3049,395.2942 | polygonatumoside F/isomer |
| 7 | 9.91 | 1227.5687 | [M-H]^-^ | C_56_H_92_O_29_ | 2.93 | 1095.5260,1065.5159,933.4723,771.4183 | polygonatumoside F/isomer |
| 8 | 11.01 | 1121.5345 | [M-H_2_O+H]^+^ | C_53_H_86_O_26_ | -2.55 | 593.3711,413.3052,395.2954 | 26-*O*-(*β*-*D*-glucopyranosyl)-furost-5-ene-3*β*,14*α*,22*α*,26-tetraol 3-*O*-*β*-*D*-glucopyranosyl-(1→2)-*β*-*D*-glucopyranosyl-(1→4)-2”-*O*-acetyl-*β*-*D*-galactopyranoside/isomer |
| 9 | 11.42 | 312.1234 | [M-H]^-^ | C_18_H_19_NO_4_ | -2.34 | 190.0502 | *N*-*trans*-feruloyloctopamine/isomer |
| 10 | 12.00 | 1239.5692 | [M-H]^-^ | C_57_H_92_O_29_ | 3.30 | 1077.5149 | (25*S*)-Kingianoside D+2Glc/isomer |
| 11 | 12.19 | 1079.5315 | [M-H]^-^ | C_51_H_84_O_24_ | 3.24 | 917.4784,755.4233 | (25*S*)-26-*O*-(*β*-*D*-glucopyranosyl)-furost-5-en3*β*,22*α*,26-trio1 3-*O*-*β*-*D*-glucopyranosyl-(1→2)-*β*-*D*-glucopyranosyl-(1→4)-*β*-*D*-glucopyranoside/isomer |
| 12 | 12.38 | 1195.5725 | [M-H_2_O+H]^+^ | C_56_H_92_O_28_ | -1.40 | 1195.5743,739.4267,415.3208,397.3101 | timosaponin H1/isomer |
| 13 | 12.40 | 1211.5732 | [M-H]^-^ | C_56_H_92_O_28_ | 2.47 | 1079.5313,1049.5203,917.4782,755.4232 | timosaponin H1/isomer |
| 14 | 12.82 | 1209.5585 | [M-H]^-^ | C_56_H_90_O_28_ | 0.00 | 1077.5162,1047.5069,915.4623,753.4079 | polygodoraside E/isomer |
| 15 | 17.20 | 1217.5552 | [M+Na]^+^ | C_56_H_90_O_27_ | -0.75 | 739.4264,577.3733,415.3206,397.3098 | polygodoside G/isomer |
| 16 | 17.51 | 329.2328 | [M-H]^-^ | C_18_H_34_O_5_ | -1.51 | 329.2327,229.1435,211.1332,183.1383,171.1021 | 11, 12, 13-trihydroxy-9-octadecenoic acid/isomer |
| 17 | 20.83 | 318.3004 | [M+H]^+^ | C_18_H_39_NO_3_ | 0.32 | 256.2630 | phytosphingosine/isomer |
| 18 | 21.15 | 1101.5071 | [M+Na]^+^ | C_51_H_82_O_24_ | -1.58 | 575.3578,413.3037,395.2947,1101.5079 | pod III/isomer |
| 19 | 21.70 | 329.1027 | [M-H]^-^ | C_18_H_18_O_6_ | -0.91 | 223.0609,208.0368,193.0142,165.0193 | (3*R*)-5,7-dihydroxy-6-methy1-8-methoxy-3-(4'-hydroxybenzyl)-chroman-4-one/isomer |
| 20 | 21.75 | 1047.5066 | [M-H]^-^ | C_50_H_80_O_23_ | 4.67 | 915.4633,885.4524,753.4085,591.3538 | (25*R/S*)-POD-II/isomer |
| **21** | 22.02 | 915.4633 | [M-H]^-^ | C_45_H_72_O_19_ | 4.15 | 755.4145,753.4078,591.3539 | (25*S*)-kingianoside D/isomer |
| **22** | 22.84 | 313.1078 | [M-H]^-^ | C_18_H_18_O_5_ | -0.96 | 207.0656,179.0707 | (3*R*)-5,7-dihydroxy-6,8-dimethy1-3-(4'-hydroxybenzyl)-chroman-4-one/isomer |
| **23** | 23.88 | 359.1136 | [M-H]^-^ | C_19_H_20_O_7_ | 0.00 | 313.1079,265.1803,239.2006 | ophiopogonanone E |
| **24** | 26.91 | 327.1809 | [M-H]^-^ | C_17_H_28_O_6_ | -1.22 | 265.1800,239.2009 | spiculisporic acid/isomer |

**Table S2. Immune cells and antibody markers**

| immune cells | Labeling antibodies |
| --- | --- |
| CD4^+^ T | CD45^+^ CD3^+^ CD4^+^ CD8^-^ |
| CD8^+^ T | CD45^+^ CD3^+^ CD4^-^ CD8^+^ |
| Naïve CD4^+^ T | CD45^+^ CD3^+^ CD4^+^ CD8^-^ CD45RA^+^ CCR7^+^ |
| Naïve CD8^+^ T | CD45^+^ CD3^+^ CD4^-^ CD8^+^ CD45RA^+^ CCR7^+^ |
| CD4^+^ TCM | CD45^+^ CD3^+^ CD4^+^ CD8^-^ CD45RA^-^ CCR7^+^ |
| CD8^+^ TCM | CD45^+^ CD3^+^ CD4^-^ CD8^+^ CD45RA^-^ CCR7^+^ |
| CD4^+^ TEM | CD45^+^ CD3^+^ CD4^+^ CD8^-^ CD45RA^-^ CCR7^-^ |
| CD8^+^ TEM | CD45^+^ CD3^+^ CD4^-^ CD8^+^ CD45RA^-^CCR7^-^ |
| CD4^+^ TEMRA | CD45^+^ CD3^+^ CD4^+^ CD8^-^ CD45RA^+^ CCR7^-^ |
| CD8^+^ TEMRA | CD45^+^ CD3^+^ CD4^-^ CD8^+^ CD45RA^+^ CCR7^-^ |
| CD28^-^ CD57^+^ CD4^+^ TEMRA | CD45^+^ CD3^+^ CD4^+^ CD8^-^ CD45RA^+^ CCR7^-^ CD28^-^ CD57^+^ |
| CD28^-^ CD57^+^ CD8^+^ TEMRA | CD45^+^ CD3^+^ CD4^-^ CD8^+^ CD45RA^+^CCR7^-^ CD28^-^ CD57^+^ |
| DN B cell  CD56⁺ CD16^+^ NK cell  CD56⁺ CD16^-^ NK cell | CD45^+^ CD3^-^ CD19^+^ CD27^-^ lgD^-^  CD45^+^ CD3⁻ CD56⁺ CD16^+^  CD45^+^ CD3⁻ CD56⁺ CD16^-^ |

**Supplementary Table S3. Hematological results of the YZG intervention group and the placebo group in the elderly population.**

| **Project** | **Con-base (Median** /**Mean))** | **Con-Day 90 (Median/** **Mean))** | ***P* value** | **YZG-base**  **(Median/Mean)** | **YZG-Day 90**  **(Median/** **Mean))** | ***P* value** |
| --- | --- | --- | --- | --- | --- | --- |
| WBC（10^9/L） | 5.80 | 5.60 | 0.4250 | 5.70 | 5.60 | 0.2408 |
| RBC（10^12/L） | 4.60 | 4.70 | 0.0054^*^ | 4.505 | 4.575 | 0.3821 |
| HCT（%） | 41.00 | 42.00 | 0.0065^*^ | 41.00 | 41.00 | 0.5010 |
| GLU（mmol/L） | 5.65 | 5.79 | 0.2273 | 5.33 | 5.51 | 0.0977 |
| UREA（mmol/L） | 4.95 | 5.19 | 0.4012 | 5.130 | 5.33 | 0.4582 |
| HbA1C（%） | 5.70 | 6.00 | 0.0270^*^ | 5.80 | 5.90 | 0.0492^*^ |
| Hb（g/L） | 141.00 | 142.00 | 0.1188 | 139.4 | 138.4 | 0.6603 |
| ALB（g/L） | 44.82 | 46.09 | 0.0062^*^ | 43.92 | 45.27 | 0.0056^*^ |
| ALT（U/L） | 23.00 | 20.00 | 0.0208^*^ | 19.00 | 18.00 | 0.1393 |
| LDL-CH（mmol/L） | 3.428 | 3.380 | 0.7170 | 3.190 | 3.242 | 0.7256 |
| TRIG（mmol/L） | 1.46 | 1.42 | 0.9579 | 1.53 | 1.40 | 0.8246 |
| HDL-CH（mmol/L） | 1.481 | 1.606 | 0.0629 | 1.431 | 1.566 | 0.0401^*^ |
| CREA（μmol/L） | 67.00 | 66.00 | 0.5451 | 75.00 | 73.00 | 0.5575 |
| UA（μmol/L） | 339.8 | 315.1 | 0.1210 | 340.5 | 308.7 | 0.0200^*^ |
| AST（U/L） | 22.00 | 20.00 | 0.0586 | 20.00 | 20.00 | 0.7828 |
| TCHOL（mmol/L） | 5.910 | 5.985 | 0.6546 | 5.571 | 5.668 | 0.6359 |
| TP（g/L） | 73.56 | 73.32 | 0.7386 | 71.28 | 72.94 | 0.0112^*^ |
| PLT（10^9/L） | 229.50 | 214.00 | 0.1311 | 213.00 | 218.00 | 0.9510 |
| M（%） | 6.00 | 5.60 | 0.0668 | 5.774 | 5.783 | 0.9709 |
| M#（10^9/L） | 0.35 | 0.31 | 0.0805 | 0.330 | 0.310 | 0.4523 |
| RDW-CV（%） | 12.75 | 12.80 | 0.9709 | 12.64 | 12.65 | 0.8768 |
| RDW-SD（fl） | 43.26 | 42.91 | 0.3244 | 42.60, | 42.50 | 0.5957 |
| INS（μU/mL） | 9.44 | 8.12 | 0.1438 | 8.51 | 6.36 | 0.0507 |
| L（%） | 33.17 | 34.56 | 0.2841 | 34.77 | 34.65 | 0.9268 |
| L#（10^9/L） | 1.90 | 2.00 | 0.8059 | 1.90 | 1.80 | 0.2651 |
| MCV（fl） | 89.89 | 89.83 | 0.9118 | 90.55 | 90.37 | 0.7514 |
| MCH（pg） | 31.00 | 30.00 | 0.0001^*^ | 31.00 | 30.00 | 0.0008^*^ |
| MCHC（g/L） | 345.00 | 336.00 | <0.0001^*^ | 342.00 | 335.00 | <0.0001^*^ |
| BA（%） | 0.50 | 0.40 | 0.6116 | 0.40 | 0.40 | 0.9113 |
| BA#（10^9/L） | 0.03 | 0.03 | 0.5015 | 0.03 | 0.02 | 0.6553 |
| EO（%） | 1.80 | 2.20 | 0.1801 | 2.40 | 2.10 | 0.6233 |
| EO#（10^9/L） | 0.11 | 0.12 | 0.3873 | 0.13 | 0.10 | 0.2989 |
| A/G | 1.60 | 1.70 | 0.0037^*^ | 1.60 | 1.60 | 0.3899 |
| GLOB（g/L） | 28.75 | 27.24 | 0.0104^*^ | 27.36 | 27.67 | 0.5948 |
| PDW（fl） | 16.20 | 16.30 | 0.9183 | 16.20 | 16.20 | 0.9154 |
| MPV（fl） | 10.50 | 10.50 | 0.9878 | 10.67 | 10.71 | 0.8373 |
| PCT（%） | 0.25 | 0.23 | 0.0904 | 0.23 | 0.23 | 0.8569 |
| N（%） | 58.34 | 57.09 | 0.3782 | 55.78 | 56.22 | 0.7397 |
| N#（10^9/L） | 3.20 | 3.20 | 0.4640 | 3.10 | 2.90 | 0.3816 |

Note: Percentage of monocytes (M), Absolute monocyte count (M#), Red blood cell distribution width - coefficient of variation (RDW-CV), Red blood cell distribution width - standard deviation (RDW-SD), Red blood cell count (RBC), Hematocrit (HCT), Fasting blood glucose (GLU), Fasting insulin (INS), Percentage of lymphocytes (L), Absolute lymphocyte count (L#), Urea (UREA), Mean corpuscular volume (MCV), Mean corpuscular hemoglobin (MCH), Mean corpuscular hemoglobin concentration (MCHC), Percentage of basophils (BA), Absolute basophil count (BA#), Percentage of eosinophils (EO), Absolute eosinophil count (EO#), Glycosylated hemoglobin (HbA1C), Hemoglobin (Hb), Serum albumin (ALB), Albumin/globulin ratio (A/G), Serum alanine aminotransferase (ALT), Serum low-density lipoprotein cholesterol (LDL-CH), Serum triglycerides (TRIG), Serum high-density lipoprotein cholesterol (HDL-CH), Serum creatinine (CREA), Serum uric acid (UA), Serum globulin (GLOB), Serum aspartate aminotransferase (AST), Serum total cholesterol (TCHOL), Serum total protein (TP), Platelet distribution width (PDW), Platelet count (PLT), Mean platelet volume (MPV), Platelet hematocrit (PCT), Percentage of neutrophils (N), Absolute neutrophil count (N#).

**Supplementary Table S4**. Statistics analysis of bacterial alpha diversity among groups

| Alpha Diversity Measurements | Terms | Estimate with 95% CI | P-value |
| --- | --- | --- | --- |
| Observed Unique Species | Baseline comparison | 6.94 (-9.16, 23.04) | 0.4 |
|  | Day90 comparison | 16.19 (-2.06, 34.43) | 0.08 |
|  | YZG increment | 19.59 (2.09, 37.09) | 0.03*^*^* |
|  | Control increment | 10.34 (-6.58, 27.26) | 0.23 |
|  | Interaction term | 9.25 (-14.95, 33.45) | 0.45 |
| Shannon Diversity Index | Baseline comparison | 0.02 (-0.3, 0.35) | 0.89 |
|  | Day90 comparison | -0.01 (-0.31, 0.29) | 0.93 |
|  | YZG increment | 0.23 (-0.12, 0.59) | 0.19 |
|  | Control increment | 0.27 (0, 0.54) | 0.05*^*^* |
|  | Interaction term | -0.03 (-0.47, 0.4) | 0.88 |
| Simpson Diversity Index | Baseline comparison | 0 (-0.03, 0.03) | 0.97 |
|  | Day90 comparison | -0.01 (-0.03, 0.01) | 0.33 |
|  | YZG increment | 0.01 (-0.02, 0.04) | 0.37 |
|  | Control increment | 0.02 (0, 0.05) | 0.04*^*^* |
|  | Interaction term | -0.01 (-0.05, 0.03) | 0.6 |
| Chao 1 Diversity Index | Baseline comparison | 7.36 (-8.74, 23.46) | 0.37 |
|  | Day90 comparison | 16.42 (-1.96, 34.81) | 0.08 |
|  | YZG increment | 18.92 (1.36, 36.48) | 0.03*^*^* |
|  | Control increment | 9.86 (-7.15, 26.87) | 0.25 |
|  | Interaction term | 9.06 (-15.25, 33.37) | 0.46 |
| Pielou Index | Baseline comparison | 0 (-0.04, 0.03) | 0.89 |
|  | Day90 comparison | -0.01 (-0.05, 0.02) | 0.43 |
|  | YZG increment | 0.02 (-0.02, 0.06) | 0.32 |
|  | Control increment | 0.03 (0, 0.06) | 0.04*^*^* |
|  | Interaction term | -0.01 (-0.06, 0.04) | 0.67 |
| ACE | Baseline comparison | 7.41 (-8.69, 23.51) | 0.36 |
|  | Day90 comparison | 16.29 (-2.05, 34.64) | 0.08 |
|  | YZG increment | 18.72 (1.2, 36.23) | 0.04*^*^* |
|  | Control increment | 9.83 (-7.17, 26.84) | 0.25 |
|  | Interaction term | 8.88 (-15.39, 33.16) | 0.47 |

***References:***

Lin, H., & Peddada, S. D. (2020). Analysis of compositions of microbiomes with bias correction. *Nat Commun, 11*(1), 3514. doi:10.1038/s41467-020-17041-7

Zhang, N., Chen, Z., Huang, M., Lu, Q., Yang, H., Xiang, J., . . . Huang, J. (2024). Cervicovaginal microbiota long-term dynamics and prediction of different outcomes in persistent human papillomavirus infection. *J Med Virol, 96*(2), e29451. doi:10.1002/jmv.29451

Zhang, X., Li, X., Xu, H., Fu, Z., Wang, F., Huang, W., . . . Yin, S. (2023). Changes in the oral and nasal microbiota in pediatric obstructive sleep apnea. *J Oral Microbiol, 15*(1), 2182571. doi:10.1080/20002297.2023.2182571
